# Climate Change Impairs Nitrogen Cycling in European Beech Forests

**DOI:** 10.1371/journal.pone.0158823

**Published:** 2016-07-13

**Authors:** Michael Dannenmann, Carolin Bimüller, Silvia Gschwendtner, Martin Leberecht, Javier Tejedor, Silvija Bilela, Rainer Gasche, Marc Hanewinkel, Andri Baltensweiler, Ingrid Kögel-Knabner, Andrea Polle, Michael Schloter, Judy Simon, Heinz Rennenberg

**Affiliations:** 1 Institute of Meteorology and Climate Research, Atmospheric Environmental Research (IMK-IFU), Karlsruhe Institute of Technology (KIT), Kreuzeckbahnstrasse 19, 82467 Garmisch-Partenkirchen, Germany; 2 Institute of Forest Sciences, Chair of Tree Physiology, University of Freiburg; Georges-Koehler-Allee 53/54, 79110 Freiburg, Germany; 3 Lehrstuhl für Bodenkunde, Department für Ökologie und Ökosystemmanagement, Wissenschaftszentrum Weihenstephan. Technische Universität München, 85350 Freising-Weihenstephan, Germany; 4 Environmental Genomics, Helmholtz Zentrum München, German Research Center for Environmental Health, Ingolstädter Landstr. 1, 85764 Neuherberg, Germany; 5 Forest Botany and Tree Physiology, Büsgen-Institute, Georg-August Universität Göttingen, Büsgenweg 2, 37077 Göttingen, Germany; 6 IAS-Institute for Advanced Study, Technische Universität München, Lichtenbergstraße 2a, D-85748 Garching, Germany; 7 King Saud University, PO Box 2454, Riyadh 11451, Saudi Arabia; 8 Chair of Forestry Economics and Forest Planning, University of Freiburg, 79110 Freiburg, Germany; 9 Research Unit Forest Resources and Management, Swiss Federal Research Institute WSL, Zuercherstrasse 111, CH-8903 Birmensdorf, Switzerland; University of California Davis, UNITED STATES

## Abstract

European beech forests growing on marginal calcareous soils have been proposed to be vulnerable to decreased soil water availability. This could result in a large-scale loss of ecological services and economical value in a changing climate. In order to evaluate the potential consequences of this drought-sensitivity, we investigated potential species range shifts for European beech forests on calcareous soil in the 21^st^ century by statistical species range distribution modelling for present day and projected future climate conditions. We found a dramatic decline by 78% until 2080. Still the physiological or biogeochemical mechanisms underlying the drought sensitivity of European beech are largely unknown. Drought sensitivity of beech is commonly attributed to plant physiological constraints. Furthermore, it has also been proposed that reduced soil water availability could promote nitrogen (N) limitation of European beech due to impaired microbial N cycling in soil, but this hypothesis has not yet been tested. Hence we investigated the influence of simulated climate change (increased temperatures, reduced soil water availability) on soil gross microbial N turnover and plant N uptake in the beech-soil interface of a typical mountainous beech forest stocking on calcareous soil in SW Germany. For this purpose, triple ^15^N isotope labelling of intact beech seedling-soil-microbe systems was combined with a space-for-time climate change experiment. We found that nitrate was the dominant N source for beech natural regeneration. Reduced soil water content caused a persistent decline of ammonia oxidizing bacteria and therefore, a massive attenuation of gross nitrification rates and nitrate availability in the soil. Consequently, nitrate and total N uptake of beech seedlings were strongly reduced so that impaired growth of beech seedlings was observed already after one year of exposure to simulated climatic change. We conclude that the N cycle in this ecosystem and here specifically nitrification is vulnerable to reduced water availability, which can directly lead to nutritional limitations of beech seedlings. This tight link between reduced water availability, drought stress for nitrifiers, decreased gross nitrification rates and nitrate availability and finally nitrate uptake by beech seedlings could represent the Achilles’ heel for beech under climate change stresses.

## Introduction

European beech (*Fagus sylvatica* L.) dominates the natural forest vegetation in moist to moderately dry areas of the sub-mountainous altitude range in Central Europe [1]. About one third of the potential beech forest area in Central Europe is located on calcareous soil that is highly susceptible to water deprivation. For future forestry in Central Europe, it has even been suggested to replace spruce by beech [2,3]. However, beech responds to lower water availability with reduced growth and competitive power, and this apparent drought sensitivity of beech is a matter of current concern and debate [4–10] due to observations of increased heat waves and drought periods in wide regions of Central Europe [11]. This trend is expected to continue and intensify in the coming decades [12,13]. Two different mechanisms have been proposed to explain the sensitivity of beech to increased temperature and drought [4–6]: (1) tree physiological limitations; and (2) nutritional limitations due to decelerated microbial N turnover in soil, specifically due to impaired liberation and provision of bioavailable N by soil microbes. The latter mechanism may be of particular importance in calcareous soils and apply especially for beech seedlings with their restricted root system, because Rendzic Leptosols are poor in bioavailable N [14,15]. Furthermore, these soils are characterized by a shallow profile, high gravel content, clayey texture, and low water and nutrient retention capacity. Hence, bioavailable N released by microorganisms is frequently limiting growth of this forest type [5,15–17]. However, due to the lack of experimental evidence, and a range of severe methodological problems in the quantification of gross N turnover in the plant-soil-microbe system, this mechanism remained a hypothesis.

Nitrogen mineralization and nitrification as well as the subsequent competitive partitioning of bioavailable organic N monomers and inorganic N compounds between the soil microbes and vegetation regulate tree N uptake in N-limited forests. There is now ample evidence that trees—including European Beech—successfully compete for organic N as well as for mineral N with free living microorganisms in a wide range of forest ecosystems with or without the help of mycorrhizal fungal symbionts [5,15,16,18]. Hence, trees can actively influence soil microbial N turnover processes by competing for the same substrates at several stages of the N cycle: (1) Trees may take up monomeric organic compounds such as amino acids thus limiting this substrate for microbial uptake of dissolved organic nitrogen (DON) and microbial ammonification. (2) Trees may take up ammonium (NH_4_^+^) thus reducing substrate availability for autotrophic nitrification and microbial immobilisation. (3) Trees may take up nitrate (NO_3_^-^) thus limiting microbial NO_3_^-^ utilization [5,18,19–22]. Furthermore, trees influence microbial N turnover in soil by the determination of organic matter composition via plant residues, thus influencing substrate quality for N mineralization [20] and by direct carbon (C) allocation to microorganisms via root exudation [5,16,20].

Surprisingly, the recognition of the importance of plant-microbe interactions for gross N turnover has only rarely been reflected in experimental designs for quantification of soil or plant N turnover. In contrast, soil scientists usually determine gross N turnover rates in plant-free disturbed soil, and plant physiologists frequently deploy N uptake measurements with washed roots in absence of soil and soil microorganisms [23]. Such experimental approaches neglect plant-soil-microbe C/N interactions such as plant-microbe competition for N, microbial resupply of plant nutrients and plant rhizodeposition effects on microbial soil N turnover [5]. Hence it may be questioned if N flux rates determined based on isolated views of the plant or soil compartments are realistic. Studies interlinking both sides are less common and have been performed aiming at elucidating plant-microbial competition for N in different ecosystems like tundra [24], alpine meadow [25], grassland [26–30] and temperate forests [31–36]. However, these studies almost exclusively performed simple tracing experiments aiming at the determination of ^15^N recovery rates in plant and microbial N pools rather than providing actual N turnover rates. Studies characterizing the competition for N between plants and microorganisms in a process-oriented way, i.e., by determining simultaneously occurring rates of microbial N turnover in soil and N uptake by plants are still very rare [5,22,37].

Thus, our goals were (1) to explore the extent of potential species range shifts of European Beech on marginal calcareous soils in the 21^st^ century and (2) to test whether there is a soil microbial mechanism limiting beech seedling N nutrition and establishment and thus competitive strength under reduced water availability in a changing climate, potentially contributing to expected species range shifts on top of plant physiological constraints. Therefore, we conducted statistical species range modelling for European beech forests on calcareous soil for present day conditions and for the year 2080. In view of the importance of seedling establishment for species range shifts we assessed beech seedling performance under exposure to climate change conditions in a field experiment, thereby considering soil microbial community composition, gross rates of microbial N turnover, soil N availability and N nutrition of beech seedlings. For this purpose, we deployed an enriched stable isotope-based experimental approach to simultaneously quantify all major N turnover processes in intact beech seedling-soil-microbe systems, thereby maintaining plant-soil microbe interactions and competition for N. By translocation of soil-mesocosms containing natural beech regeneration across a narrow valley from the northwest (NW, cool moist microclimate) to the southwest (SW, warm-dry microclimate) aspect, we combined this approach with a space-for-time climate change experiment. In addition to exposure-induced reduction of soil water availability, a roof system accelerated drought during a 39 days period at the SW aspect. Supporting measurements included abundance of genes related to ammonia oxidation, mycorrhizal colonization and N metabolite levels in fine roots as well as plant biomass and isotope recovery in beech seedlings three months after ^15^N isotope labelling.

## Materials and Methods

### Statistical species distribution model

For the species distribution model we used presence/absence information for European beech derived from an international monitoring network (ICP level I—more than 8000 plots with 1097 presence observations of European beech) as response variable that we combined with high-resolution derivations of precipitation and temperature from the WorldClim- database, which implies modelling the realized niche of the species. For the projection under future conditions, we used output from the global circulation model HADCM3 driven by the SRES scenario A2 [38] until the year 2080, which was calibrated and statistically downscaled to 30-arc-second tiles. We fitted a Generalized Linear Model (GLM) with logit link function and calibrated the model by a stepwise selection using the Bayesian Information Criterion (BIC) with five bioclimatic variables entering the model including: yearly sum of degree days above 5°C, iso-thermality, a yearly drought index, sum of precipitation in the warmest quarter of the year and the precipitation of the most humid month. As the threshold value for transforming predicted probabilities into binary presence information we used Cohen’s Kappa. We used the area under curve (AUC) of the receiver operating characteristic (ROC) and Cohen’s Kappa to evaluate the performance of the model. Both, AUC with a value of 0.86 and Kappa with a maximum value of 0.43, indicate good performance of the model (S1 Fig). To validate the model we carried out a 10-fold cross-validation, for which we randomly split the data into a training and an evaluation dataset. The model appears to be robust with the cross validation means being very close to the model values and the variation within cross validation results being low (S1 Table). The species distribution maps for current and projected climate were intersected with a map of the Geology of Europe depicting calcareous and limestone substrates. More detailed information on the statistical species distribution modelling is provided in the Supplementary Materials and Methods.

### *In situ* climate change experiment: site description and experimental design

#### The Tuttlingen experimental beech forest

The *in situ* climate change experiment with its biogeochemical, microbiological and plant physiological field studies was based on intact plant-soil-mesocosms with beech seedlings and was conducted in a typical N-limited mountainous beech forest growing on a Rendzic Leptosol in Southern Germany (Tuttlingen experimental beech forest, 8°45´E; 47°59´N, ca. 800 m a.s.l.). Mean annual air temperature is approximately 6.5°C and the average annual rainfall amounts to 854 mm (1961–1990). The clay-rich soil is classified as Rendzic Leptosol (Skeletic) according to the International Union of Soil Sciences Working Group WRB (2007) derived from horizontally bedded limestone and marls. Soil profiles are shallow followed by weathered parent rock containing > 45% gravel and stones. Due to nutrient poor soils and low atmospheric N input, soil N cycling is characterized by competitive partitioning of N between beech and associated mycorrhiza vs. free living soil microorganisms [14–16]. Permits for the experiment were issued by the Landratsamt Tuttlingen, Germany.

#### Experimental design

**Fig 1:**
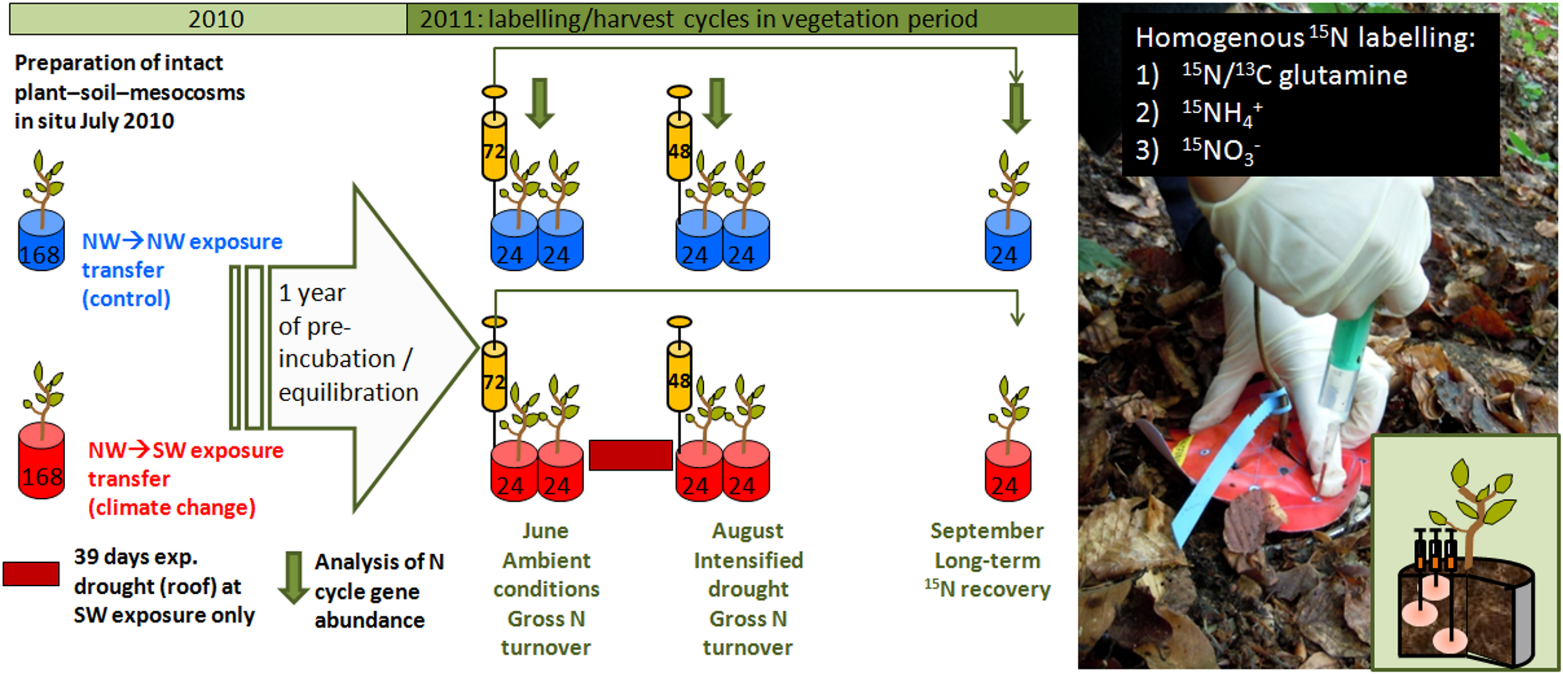
Experimental design. The figure illustrates coring of beech-soil-mesocosms by use of stainless steel cylinders with subsequent pre-incubation for one year either under cool-moist microclimate at the coring site (NW exposure, control) or warm-dry microclimate (SW-exposure, climate change). After pre-incubation and equilibration, homogeneous labelling of the intact beech seedling-soil-microbe systems with either ^15^N/^13^C-enriched glutamine, ^15^N-ammonium (NH_4_^+^) or ^15^N-nitrate (NO_3_^-^) and subsequent double harvests (6 and 48 hours after labelling) were conducted for determination of gross N turnover rates in the beech seedling-soil-microbe system in June (comparison of ambient NW vs. SW climatic conditions) and August (ambient NW conditions vs. roof-intensified drought at SW). A final sampling of mesocosms labelled in June allowed investigating long-term isotope recovery in September (three months after labelling). All three sampling dates were accompanied by determination of supporting soil and plant parameters such as abuncance of microbial genes related to ammonia oxidation, mycorrhization, and plant metabolites.

The climate change treatment was established using a space-for-time approach and combined with isotope-based process studies (Fig 1). We used topographic variability as a model for climate variability, thus identifying model ecosystems for present day and future climate conditions located on opposing slopes of a narrow valley. The control site with its NW aspect and cool-moist microclimate (representing current climate conditions) is located at a distance of less than 1 km to the climate change site with its SW aspect and warm-dry microclimate, representing “future climate conditions”. Microclimate at SW exposure is characterized by increased daily maximum of air and topsoil temperatures and thus reduced water availability [14]. Therefore, the SW aspect is considered to constitute a model ecosystem with local climatic patterns equalling the climatic conditions predicted for coming decades [39]. Climate change was simulated by transferring stainless steel cylinders (15 cm height, 16.4 cm inner diameter) containing intact beech seedling-soil-microbe mesocosms of uniform size from NW exposure to SW exposure, with control transfers at the NW exposure sampling site [40].

The incubation areas were in fenced zones of 400 m^2^ either in immediate vicinity to the sampling area (NW) or at the SW slope in 1 km distance characterized by the same inclination. Both NW and SW incubation areas showed closed canopy and only beech trees were present at a distance of 50 m. After full development of leaves, photosynthetic active radiation levels at the forest floor of the research site are mainly regulated by the canopy rather than by slope exposure [3]. The simulated climate change conditions at the SW slope were intensified through a temporal (June 27, 2011—August 05, 2011) rain-out sheltering roof approximately 1 m above ground level (Fig 1). All mesocosms incubated at SW exposure were under the roof in this period.

After an equilibration period of one year, short-term-isotope-tracing-based process studies were conducted via soil ^15^N labelling followed by destructive sampling to simultaneously quantify all relevant gross N turnover processes in the plant-soil-microbe system. Labelling of beech-soil-microbe-mesocosms took place on June 22, 2011 (72 beech-soil-mesocosms at NW and SW each), to (1) compare gross N turnover between ambient conditions at SW and NW exposure based on full destructive sampling six hours and 48 hours after labelling, and (2) to investigate ^15^N recovery at the end of growing season through a final sampling in September. A second labelling campaign took place after the roof period on August 2 (48 beech-soil-mesocosms at NW and SW each), followed again by sampling six and 48 hours after labelling. The August labelling/sampling cycle allowed to compare gross N turnover between ambient conditions at NW and roof-intensified drought (39 days) conditions at SW. Isotope recovery in the plant-soil-N pools three months after labelling was investigated in a third, final sampling in September 2011 (Fig 1). At all three sampling dates, supporting soil, microbial, mycorrhizal and plant parameters as well as abundance genes encoding for ammonia monooxygenase in soil were determined as described in the following sections.

#### Coring and transfer of intact beech seedling-soil-mesocosms

Overall, 256 ^15^N-labelled and 32 unlabelled beech seedling-soil-mesocosms were analysed in this study. Coring and Transfer of beech seedling-soil-mesocosms was conducted within one week in absence of precipitation in July 2010. For coring, a suitable sampling area of 50 * 50 m with identical slope (18°) and similar soil profile was identified. In this representative area, finer grained soil (Ah horizon) was only found in the uppermost 15–20 cm (i.e., the main rooting zone of beech natural regeneration). There was a sharp transition between this almost gravel-free clay-rich finer grained soil and the deeper gravel-dominated periglacial layers which were not suitable for coring. In this sampling area, beech natural regeneration trees of 2.2–2.5 mm stem diameter and 30–40 cm height were selected. Custom-made stainless steel cylinders (height 150 mm, outer diameter 168 mm, sharp edge at bottom, wall thickness 2 mm, open at bottom and top) were manually driven into the soil with the selected beech seedling growing in the centre. A custom-made extension tool fitting to the stainless steel cylinders facilitated coring under avoidance of damage to the aboveground part of the plant. After coring, the cylinders, containing an intact beech-soil-mesocosm, were carefully excavated under preservation of an even ending at the bottom. Excavated beech-soil-mesocosms were immediately reburied vertically either within NW or after transfer to SW. The litter layer was preserved and contained in the cylinders during coring. Pre-tests showed that the entire root system of beech seedlings of this size fitted well to the embraced volume of the used steel cylinders. Only mesocosms without visible damage or cut beech roots were used.

While reburying beech-soil-mesocosms, extreme care was taken to create a realistic transition between the bottom of the soil cores and the ambient soil. For this purpose, beech-soil-mesocosms were placed vertically in holes with a 5 cm thick horizontal bed made of homogenized NW soil and subsequently gently hammered down 2 cm at the new position. Finally, continuous contact between the litter layer on the transferred beech-soil-mesocosms and the surrounding litter layer at the new position was established. After transfer, all reburied intact beech-soil-mesocosms at both sites were irrigated with 500 ml of water over a period of two hours to avoid drying or death of enclosed beech seedlings following transfer. The amount of water corresponded to a precipitation event of 23.7 l m^-2^, i.e., a typical summer convective rainfall event. The survival rate of transferred beech seedlings was 97%.

Ten further beech-soil-mesocosms were transferred (5 within the NW slope, 5 to the SW slope) to monitor soil temperature and soil moisture in 5 cm depth in the mesocosms (n = 5 each slope) using horizontally installed combined soil moisture/temperature probes (DECAGON EC-5, Decagon Devices, Inc., Pullman, USA) with an hourly temporal resolution.

### Isotope labelling of beech seedling-soil-mesocosms

In order to facilitate the simultaneous quantification of gross rates of all major N turnover processes in the beech-soil-microbe system, the intact soil-root-mesocosms were labelled with either ^15^N/^13^C-enriched glutamine, ^15^NH_4_^+^ or ^15^NO_3_^-^. To enable homogeneous distribution of isotopes in the root-soil system, we applied an isotope injection method developed for large soil cores [41] and optimized and adapted it for the Tuttlingen soil. For this purpose, we conducted pre-experiments in March 2011 using Brilliant Blue FCF colour dye instead of ^15^N enriched label solution. Various injection patterns and depths, numbers of injections per mesocosm and solution volumes per single injection were tested to achieve homogeneous 3-dimensional distribution of label solution in the soil-root-system at minimized amounts of injected liquid to minimize label artefacts such as stimulation of N turnover and leakage of labeled solution at the bottom of the beech-soil-mesocosms. In this pre-experiment, mesocosms were harvested one day after dye application by removing soil stepwise from bottom to top in layers of 2 cm. The distribution of the colour dye in the soil was monitored visually. Outflow at the bottom of the labelled beech-soil-mesocosms was examined by storing the beech-soil-mesocosms on white paper sheets. To inject dye and later ^15^N solution we used custom-made stainless steel side port cannulas with lasered depth check marks. In this labelling optimization experiment the following setup facilitated homogeneous colour dye distribution with only minimal leakage: 16 single amounts of 3 ml solution each were injected into the beech seedling-soil-mesocosms to a depth of 1 and 3 cm each, and another 8 injections of 3 ml each to a depth of 6 cm. Additionally, 10 ml of ^15^N-labelled solution were homogeneously spread on top of the soil surface. Hence, the total amount of added label solution was 130 ml, applied to 2.3 kg dry soil contained on average in the beech-soil-mesocosms. Paper calibres indicating injection patterns and depths were constructed to fit onto the stainless steel cylinders in order to ensure reproducible labelling. Before ^15^N-labelling, the litter layer was removed from the top of the soil surface and later replaced. An earlier study showed that the litter layer is of very minor significance for N nutrition of beech natural regeneration [42].

Labelling was conducted simultaneously at NW and SW starting at 5:00 am and took 3 hours (June) and 2 hours (August). The label solution always contained all three N compounds used in this experiment, i.e., NH_4_^+^, NO_3_^-^ and glutamine, and depending on the labelling treatment, either NH_4_^+^, NO_3_^-^ or glutamine was isotopically enriched (50 atom % ^15^N). Glutamine was also enriched in ^13^C (50 atom%) ^13^C for glutamine). Glutamine was chosen as an organic N compound because earlier studies showed high glutamine uptake capacities of and abundance in beech seedlings and adult beech trees at this site [15,16]. The amount of N added via label solution equalled to 3.5 mg NH_4_^+^-N kg^-1^ soil dry weight (sdw), 3.5 mg NO_3_^−^-N kg^-1^ sdw, and 0.7 mg glutamine-N kg^-1^ sdw. Soil NH_4_^+^ background concentrations in unlabeled soil were hardly different between NW and SW. However, background soil NO_3_^-^ concentrations were generally much smaller at SW than at NW. Therefore, the ratio of added NO_3_^−^-N via label solution to background soil NO_3_^-^ was larger for SW than for NW. In June, N added via label solution equalled to 76% and 80% of ambient NH_4_^+^ concentrations and 35% and 78% of ambient NO_3_^-^ concentrations at NW and SW, respectively. In August, this equalled 84% and 114% of ambient soil NH_4_^+^ concentrations. However, NO_3_^-^ levels were extremely low at SW, so that added label equalled to 211% and 762% of ambient NO_3_^-^ concentrations at NW and SW, respectively. Hence, several-fold reduced soil NO_3_^-^ levels were observed both in unlabelled mesocosms and in labelled mesocosms of the climate change treatment.

Mean total ^15^N isotope recovery (sum of extractable and non-extractable soil N pools plus plant N pools) six hours after isotope application was 74±4% (standard error of the mean) after ^15^N glutamine labelling, 86±5% after ^15^NH_4_^+^ labelling, and 61±4% after ^15^NO_3_^-^ labelling.

### Beech-soil-mesocosm harvest and sample preparation

Eight intact beech-soil-mesocosms were harvested every sampling point for each of the three N-compound labelling treatments and each of the two slopes (NW exposure, control treatment and SW exposure, climate change treatment). Consequently, 48 beech-soil-mesocosms each were sampled on June 22, 2011 (6 hours after labelling), on June 24, 2011 (48 hours after the labelling), on August 2, 2011 (6 hours after labelling) on August 4 (48 hours after labelling) and on September 27, 2011 (3 months after labelling). The harvests on June 22–24, August 2–4 and September 27 were accompanied by additional sampling of unlabelled beech-soil-mesocosms to analyse ambient background inorganic N concentrations and isotopic natural abundance of investigated pools with 4–8 replicates per exposure.

For harvesting, beech-soil-mesocosms were excavated under maintenance of an even ending at the bottom and entirely harvested within two hours after excavation. The beech seedling was cut and further processed as described below. The remaining soil/root system was carefully separated by hand into soil, gravel, dead coarse organic material and living fine and coarse roots. Additionally, mycorrhizal root tips and a subsample of rhizosphere soil (defined as soil adhering to root after vigorous shaking) were sampled and further processed as described below.

The soil contained in each mesocosm was immediately homogenized by manual mixing for 10 minutes to assure full mixing to a homogeneous sample. All soil extraction steps for analysis of N compounds and their ^15^N enrichment in soil were immediately conducted during harvest in the field lab with field fresh soil (see below). A subsample of ca. 100g soil was air dried. For nucleic acid analysis, other subsamples of bulk soil as well as the rhizosphere soil were immediately frozen at -80°C.

Fresh weight of the entire soil contained in the beech-soil-mesocsoms and the weight of the stainless steel cylinders were recorded. Gravimetric soil water content was determined with a subsample of approximately 400-500g of soil by drying at 105°C until constant weight.

During each harvest, beech seedlings were carefully removed from mesocosms and separated into leaves, stems, coarse (>2 mm diameter) and fine roots (<2 mm diameter). Remaining adhering small soil was carefully washed from the roots and dried. The fresh weight of each plant tissue was determined. Samples were dried at 60°C until constant weight. After determination of the fresh weight, fine root samples were separated into two parts for subsequent analyses: one part for ectomycorrhizal analyses was wrapped in wet tissue and stored in plastic bags at 4°C until return to the laboratory, the second part was dried at 60°C for 2 days for later EA-IRMS analyses. For biochemical analyses of N metabolites in fine roots, additional samples were taken from unlabelled beech-soil-mesocosms on June 22 and shock-frozen in liquid N until sample analyses to avoid degradation of the metabolites.

### Soil analyses

#### Total organic carbon (C), total C and N and δ^15^N in bulk soil

Soil samples were immediately frozen and freeze-dried after sampling. After removing roots and gravel using tweezers, bulk soil samples were finely ground. Determination of total soil C, N and δ^15^N was performed in duplicate for every sample with an isotope ratio mass spectrometer (Delta V, Thermo Electron Corporation, Dreieich, Germany) coupled to an elemental analyser (Euro EA, Eurovector, Milan, Italy) at the Helmholtz Zentrum München, German Research Center for Environmental Health, Institute of Soil Ecology in Neuherberg. The ^15^N atom% excess enrichment was calculated by subtracting natural abundance values gained by the unlabelled mesocosms from the values obtained of the labelled soil-mesocosms.

#### Analyses of extractable soil N pools

A representative subsample of 100 g of homogenized soil out of every beech-soil-mesocosm was immediately extracted in the field with 0.5 M K_2_SO_4_ at a soil:solution ratio of 1:1.5. Addition of K_2_SO_4_ solution was followed by 1 hour of shaking at 240 rotations per minute. After shaking, extracts were vacuum filtered using pumps and glass fibre filters [15]. Subsamples of the extract were transferred into different tubes and immediately frozen in the field for later analysis of (1) total organic C and total N concentrations, (2) NH_4_^+^ and NO_3_^-^ concentrations, (3) ^15^N enrichment in NH_4_^+^, NO_3_^-^ and dissolved organic N (DON), and (4) ^15^N enrichment in total dissolved (organic + mineral) N.

A second soil subsample was not immediately extracted, but first underwent a chloroform-fumigation over 24 hours as described in detail in an earlier publication [15] and was extracted afterwards as described above with subsequent freezing of the extract for later analysis of (1) total N (organic + organic) and (2) δ^15^N in total N. Ammonium and NO_3_^-^ concentrations in extracts were analysed colorimetrically by a commercial laboratory (Dr. Janssen, Gillersheim, Germany). Total organic C (TOC) and total N (TN) in extracts were quantified using an Infrared TOC analyser with a coupled chemoluminescence-based total N module (DIMATEC GmbH, Germany) [15]. Dissolved organic N was calculated as the difference between total N and mineral N in extracts.

The ^15^N enrichment in soil NH_4_^+^, NO_3_^-^ and DON was quantified by sequential diffusion steps, based on conversion of the target compounds into NH_4_^+^, pH increase to induce volatilization as NH_3_, and subsequent trapping of NH_3_ on acid traps prepared for isotope ratio mass spectrometry (IRMS) at the Center of Stable Isotopes of KIT-IMK-IFU as described in detail in earlier publications [41,42]. Microbial biomass N and ^15^N were quantified following the chloroform-fumigation extraction approach as described in detail in earlier publications [41,42]. No conversion factors (k_EN_) were used to render estimates of rather the active part of microbial biomass and newly immobilised N [42]. Total non-extractable soil N and the respective ^15^N recovery were calculated as the difference between total soil N and all extractable N compounds (i.e., NH_4_^+^, NO_3_^-^, DON and microbial biomass N).

### Molecular analysis of ammonia monooxyygenase gene abundance

#### Nucleic acid extraction from bulk soil and rhizosphere soil

DNA was extracted from 0.4 g bulk soil and 0.1 g rhizosphere soil, respectively, using the FastDNA^™^ SPIN Kit for Soil (MP Biomedicals, Heidelberg, Germany) and the Precellys 24 Instrument (Bertin Technologies, Montigny-le-Bretonneux, France). Quantity and quality of the extracted DNA were checked with a spectrophotometer (Nanodrop, PeqLab, Erlangen, Germany) and gel electrophoresis. The extracts were stored at -80°C until real-time PCR analysis.

#### Quantitative real-time PCR assay to quantify marker genes for ammonia oxidation

Quantitative real-time PCR (qPCR) was performed using an ABI 7300 Cycler (Life Technologies, Darmstadt, Germany) with the following assay reagents: dimethyl sulfoxide (DMSO) and bovine serum albumin (BSA) (Sigma Aldrich, Taufkirchen, Germany), primers listed in S2 Table (Metabion, Martinsried, Germany) and 2x Power SYBR Green master mix (Life Technologies, Darmstadt, Germany). The respective reaction mixtures (total volume 25 μl) for the quantification of archaeal (AOA) and bacterial (AOB) genes encoding for ammonia monooxygenase (S2 Table) consisted of: 12.5 μl SYBR Green master mix, 5 pmol of each primer, 0.5 μl 3% BSA and 2 μl DNA template.

For quantification, standard curves were calculated using serial dilutions (10^1^ to 10^6^ gene copies μl^-1^) of plasmid DNA containing PCR products of the respective genes (S2 Table). According to manufacturer’s instruction, the PCR detection limit was assessed to 10 gene copies. In advance, the optimal dilution for each amplification assay was determined by dilution series of randomly chosen DNA extracts, in order to prevent PCR inhibition. The qPCR assays were performed in 96-well plates (Life Technologies, Darmstadt, Germany) for all target genes (S2 Table). All PCR runs began with a hot start at 95°C for 10 minutes. After each run, the specificity of the SYBR Green-quantified amplicons was checked by melting curve analysis and gel electrophoresis. The amplification efficiency was calculated from the formula Eff = [10^(-1/slope)^-1] and resulted in the following average efficiencies (standard deviation less than 5% of mean) for the different genes: AOA, 89%, AOB, 97%.

### Analysis of ectomycorrhizal colonization

During harvest in the field laboratory, precleaned fine root samples were placed in tap water filled petri dishes under a dissecting microscope (Stemi SV 11; Zeiss, Jena Germany) and were thoroughly cleaned from adhering soil particles using fine forceps.

Back in the laboratory randomly chosen, 2–3 cm long parts of the root system were used for examination under a dissecting microscope (205 FA; Leica, Wetzlar, Germany) and processed according to the method of Pena et al. [43]. In each sample, 300 vital root tips were counted recording simultaneously the number of dead root tips. Occasionally, samples contained less than 300 living root tips. The root tips were classified as mycorrhizal, non mycorrhizal and dry root tips.

Samples of ectomycorrhizal root tips were collected for isotope measurements. For determination of C and N content as well as ^15^N and ^13^C enrichment, root tips were dried for three days at 60°C. 1–5 mg of root tip material was weighted in 5 x 9 mm tin capsules (IVA Analysetechnik, Meerbusch, Germany) with a micro balance (Supermicro S4; Sartorius, Göttingen, Germany). Measurements were conducted at the service unit KOSI (Kompetenzzentrum für Stabile Isotope, University Göttingen, Germany) using an isotope ratio mass spectrometer (IRMS Delta Plus, Finnigan MAT, Bremen, Germany) coupled via an interface (Conflo III, Finnigan MAT, Bremen, Germany) to an elemental analyser (NA1110, CE-Instruments, Rodano, Milano, Italy). Acet anilide was used as standard, IAEA N1 (δ^15^Nair 0.4 ‰) und N_2_ (δ^15^Nair 20.3 ‰) for N calibrations and IAEA 600 (δ^13^CVPDB -27.7 ‰) for C calibrations.

### Analysis of plant tissues

#### Total N, δ^15^N and δ^13^C in beech tissues

To determine total N, as well δ^15^N and δ^13^C notation in plant tissues, oven-dried (48h, 60°C) samples were ground to a fine powder using a ball mill (Retsch MM 100, Retsch GmbH, Haan, Germany) for leaves and fine roots, and liquid N for stems and coarse roots. Aliquots of 1.0–2.5 mg for each tissue were weighed into tin capsules (IVA Analysentechnik, Meerbusch, Germany). Samples were analysed using an elemental analyser (Vario EL, elementar Analysensysteme GmbH, Hanau, Germany) coupled via an interface (Conflow III, Finnigan MAT, Bremen, Germany to an isotope ratio mass spectrometer (Delta Plus, Thermo Finnigen MAT GmbH, Bremen, Germany). Working standards (glutamic acid), calibrated against primary standards USGS 40 (glutamic acid δ^13^CPDB = -26.39) and USGS 41 (δ^13^CPDB = 37.63) for δ^13^C and USGS 41 (δ^15^N_air_ = 47.600) for δ^15^N, were analysed after every twelfth sample to enable correction for the drift of isotopic analyses over time if required.

#### Metabolites: quantification total soluble protein, total amino acids and NO_3_^-^ in the fine roots of beech seedlings

For biochemical analyses of N metabolites in the fine roots of beech seedlings, only fine roots from unlabelled samples were used from three time points (see above). Total amino acids in the fine roots were extracted [44] from frozen homogeneous fine root material (~ 50mg) in 1 mL methanol:chloroform (3.5:1.5, v:v) and 0.2 mL buffer (pH 7.0) containing 20 mM Hepes, 5 mM EGTA and 10 mM NaF. Total amino acid concentration was quantified photometrically (Beckman Coulter Inc., Fullerton, CA, USA) at 570 nm as described by Liu et al. [45] using the colour reaction with ninhydrin reagent. Glutamine was used as a standard (Sigma, Hamburg, Germany). Total soluble proteins were extracted from (~ 50mg) frozen and ground fine root material in 1 mL buffer (1 mM EDTA, 50 mM Tris-HCl (pH 8.0), 1 mM phenylmethylsulfonyl fluoride (PMSF), 15% glycerol (v:v), 5 mM dithiothreitol (DTT) and 0.1% Triton-X 100) as previously described in detail [15]. Concentrations of total soluble protein were quantified photometrically at 595 nm using Bradford reagent (Ameresco Inc., Solon, Ohio, USA) in a UV-DU650 spectrophotometer (Beckman Coulter Inc., Fullerton, CA, USA). Bovine serum albumine (BSA A-6918, Sigma Aldrich Chemie GmbH, Taufkirchen, Germany) was used as standard. Nitrate was extracted from c. 50 mg tissue in 0.1 g washed polyvinylpyrrolidone (PVP Sigma-Aldrich Inc., Steinheim, Germany) [15]. Nitrate concentrations were determined using an ion chromatograph (DX 120, Dionex, Idstein, Germany) combined with an autosampler (AS 3500, Thermo Separation Products, Piscataway, USA) and equipped with the PeakNet software package (version 4.3, Dionex, Idstein, Germany). Nitrate, phosphate, and sulphate were used as standards.

### Calculation of N pools, isotope recovery and gross rates of N turnover

Measurements of N pool size in soil and plant as well as isotopic information of plant, soil organic, inorganic, microbial and mycorrhizal N pools was used to calculate (1) gross rates of ammonification and nitrification using isotope pool dilution approaches based on the data gained 6 hours and 48 hours after ^15^N labelling, (2) plant and microbial uptake of glutamine, NH_4_^+^ and NO_3_^-^N using ^15^N tracing approaches for the first 6 hours after ^15^N labelling, and (3) ^15^N recovery in the investigated N pools. Formulas and further details are provided in the Supplementary Materials and Methods.

### Statistics

Experimental data are shown as mean values with standard errors (SE) of the mean, if not otherwise stated. Data were log-transformed if necessary to meet the requirement of normality and subsequently analysed using two-way ANOVA with the factors exposure (NW versus SW exposure, i.e., control and climate change treatments), time and their interaction. Durbin-Watson test statistics revealed absence of autocorrelation between time points. Depending on the parameters investigated, the factor time has different levels with independent observations, since samples were taken from different beech-seedling-soil-mesocosms. There were two levels for gross rates of N turnover (June, September), three levels for N pools and abundance of microbial ammonia monooxygenase genes (June, August, September) and five levels for isotopic composition of C and N pools (June_6 hours after labelling; June_48 hours after isotope labelling; August_6 hours after isotope labelling; August_48 hours after isotope labelling and September_3 months after isotope labelling). Differences between the levels of the time factor within each treatment level were further tested by applying the Tukey post-hoc test. For plant metabolites in fine roots, single comparisons between SW and NW were performed with non-parametric U-tests due to smaller number of replicates.

## Results

### Potential species range distribution modelling for beech on calcareous soil

**Fig 2:**
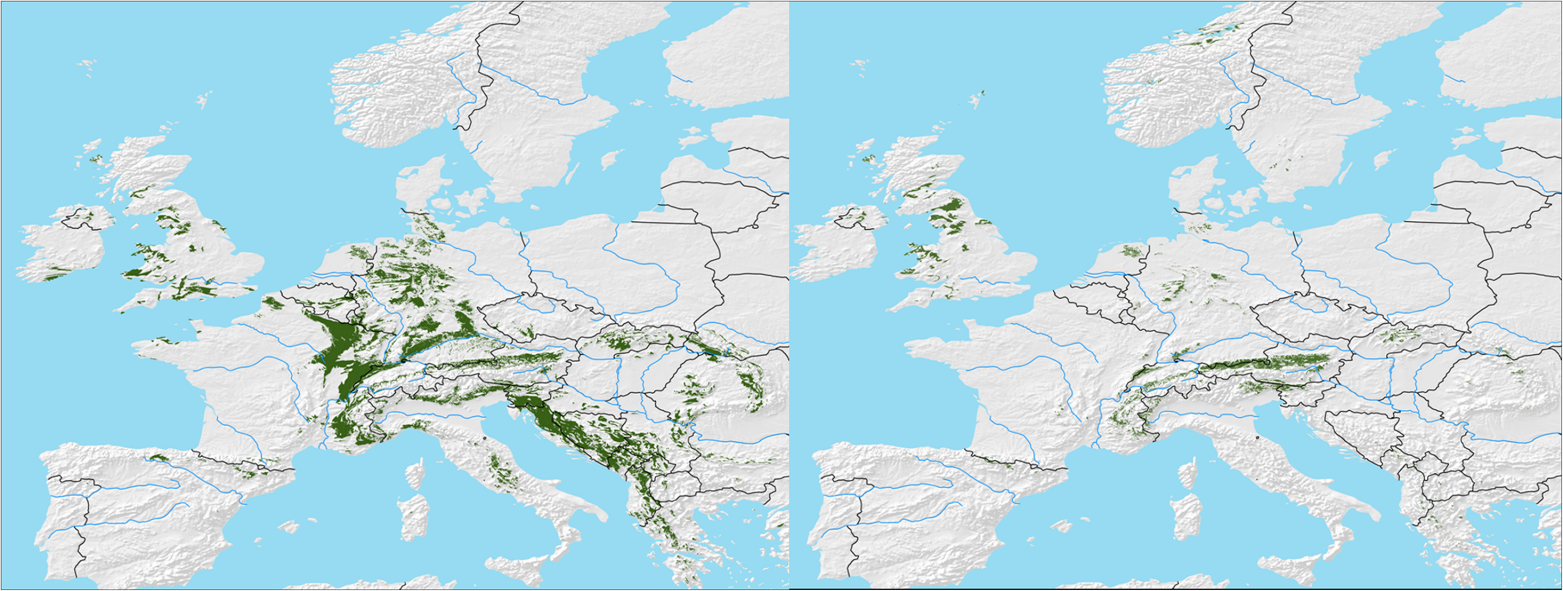
Modelled potential distribution of beech forests on calcareous soils in Europe (green colour) under current climatic conditions (left panel). I.e., For the SRES A2 scenario, we computed a potential distribution of 7.2 million ha in the year 2080 (right panel), i.e., a reduction to 22% of the current distribution. Made with Natural Earth under CC0 license.

Based on statistical species distribution models driven by climatic predictors [46,47], we computed the distribution range in the form of the realized niche of beech forests on calcareous soil in Europe. For present day conditions, the modeled potential species range distribution of European beech (Fig 2, left panel) reflected well the distribution of limestone bedrock geology. East of the Rhine rift valley, distribution patterns followed the Jurassic limestone mountain ranges along the Swabian and Franconian Cuesta Mountains from SW to SE Germany. West of the Rhine valley, there was a continuation of distribution patterns following the similar Jurassic bedrock geology towards the edge of the Paris-Seine Basin. The Jurassic mountain ranges along the border between France and Switzerland were identified to be another important potential distribution area. The Southern distribution limit was ranging from the Cantabrian Mountains and Pyrenees in Spain to the Provence in Southern France, the calcareous Apennine Mountains in Central Italy to the Balkan Mountains and the mountainous regions of Greece.

Overall these forests would cover an area of 31.4 million ha, i.e., approximately one third of the current potential beech forests in Europe (104.0 million ha), [47]). For the SRES A2 scenario [38] (Fig 2, right panel) we computed a potential distribution of 7.2 million ha in the year 2080, i.e., a drastic reduction to 22% of the current distribution. In this scenario, potential beech species range distribution is mainly reduced to Alpine regions and Great Britain with only residual spots in Northern and SW Germany (Fig 2, right panel).

### *In situ* climate change experiment

**Fig 3:**
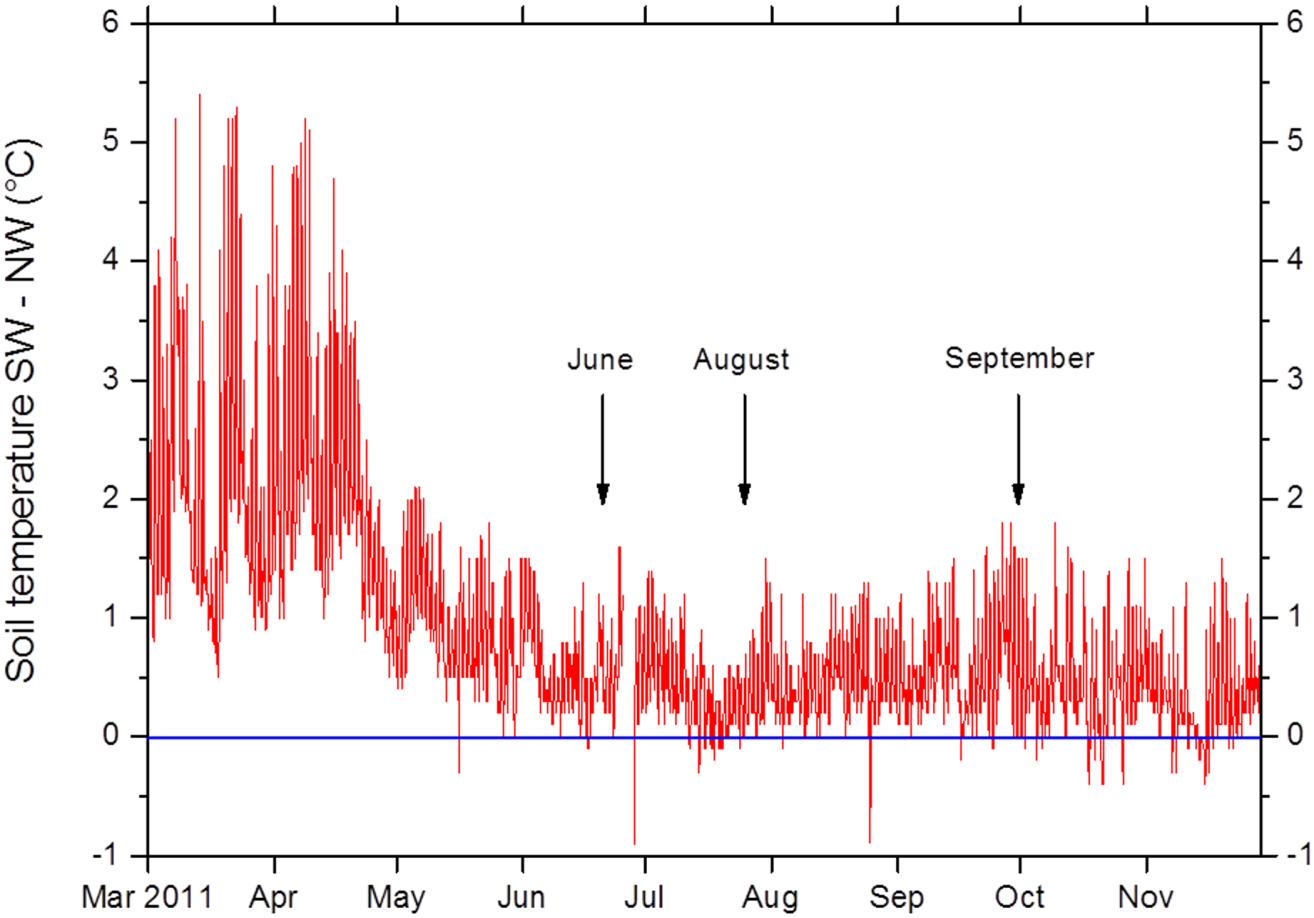
Soil temperature differences (5 cm depth) between beech-soil-mesocosms incubated at SW exposure (warm-dry microclimate, climate change treatment) and at NW exposure (cool-moist microclimate, control treatment). Data represent mean values of five temperature probes per treatment directly installed horizontally in soil of transferred beech-soil-mesocosms. Arrows indicate the three sampling campaigns. The period between the sampling in June and August equals the roof period of 39 days.

**Fig 4:**
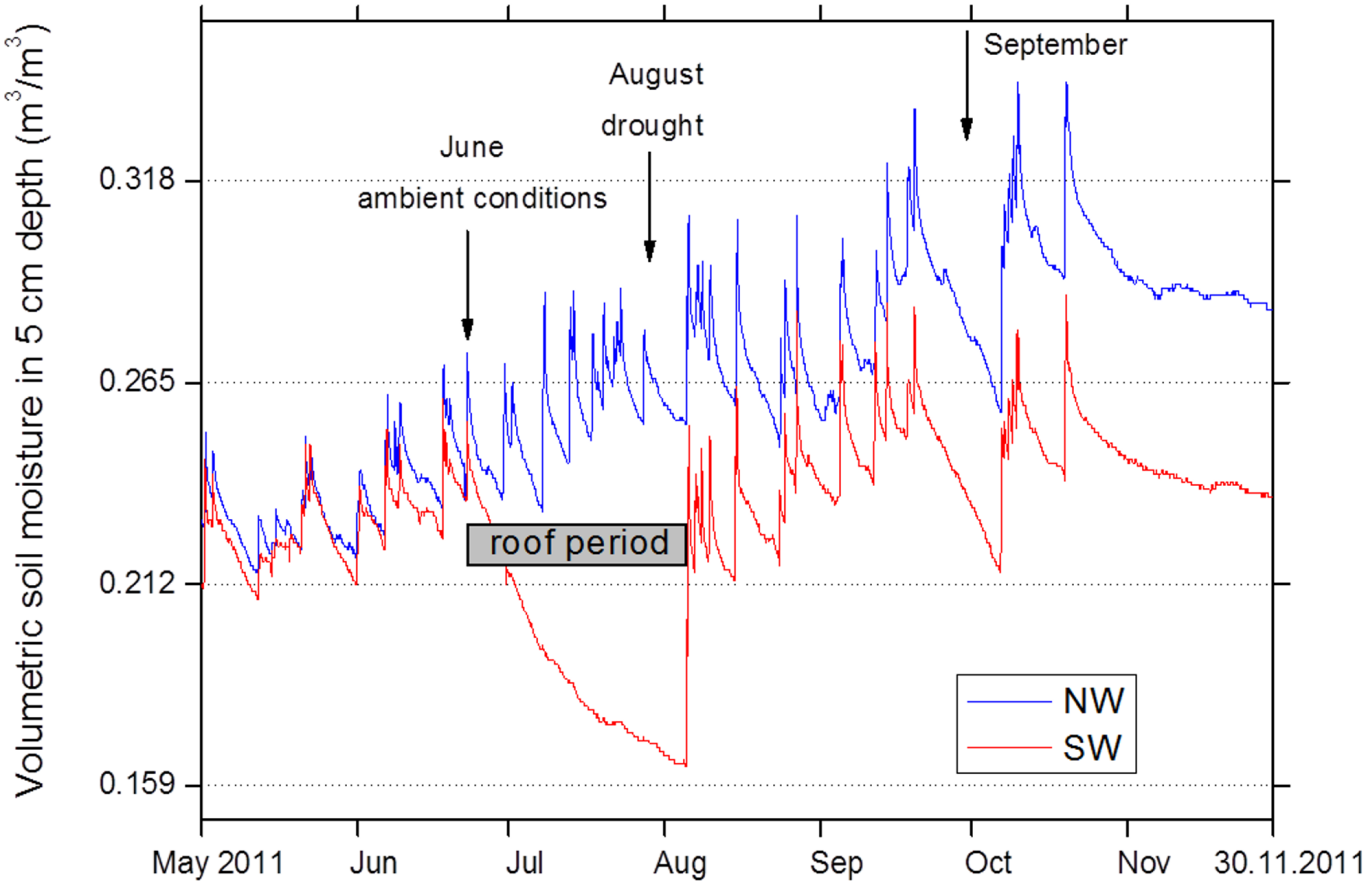
Dynamics of volumetric soil moisture in 5 cm depth (mean values of n = 5 measurements) in intact beech-soil-mesocosms of the control treatment (NW exposure, cool-moist microclimate) and climate change treatment (SW exposure, warm-dry microclimate) in the growing season 2011, i.e., 1 year after implementation of treatments by transferring beech seedling-soil-mesocosms within NW exposure or to SW exposure in summer 2010. Arrows indicate sampling campaigns (see Fig 1).

**Fig 5:**
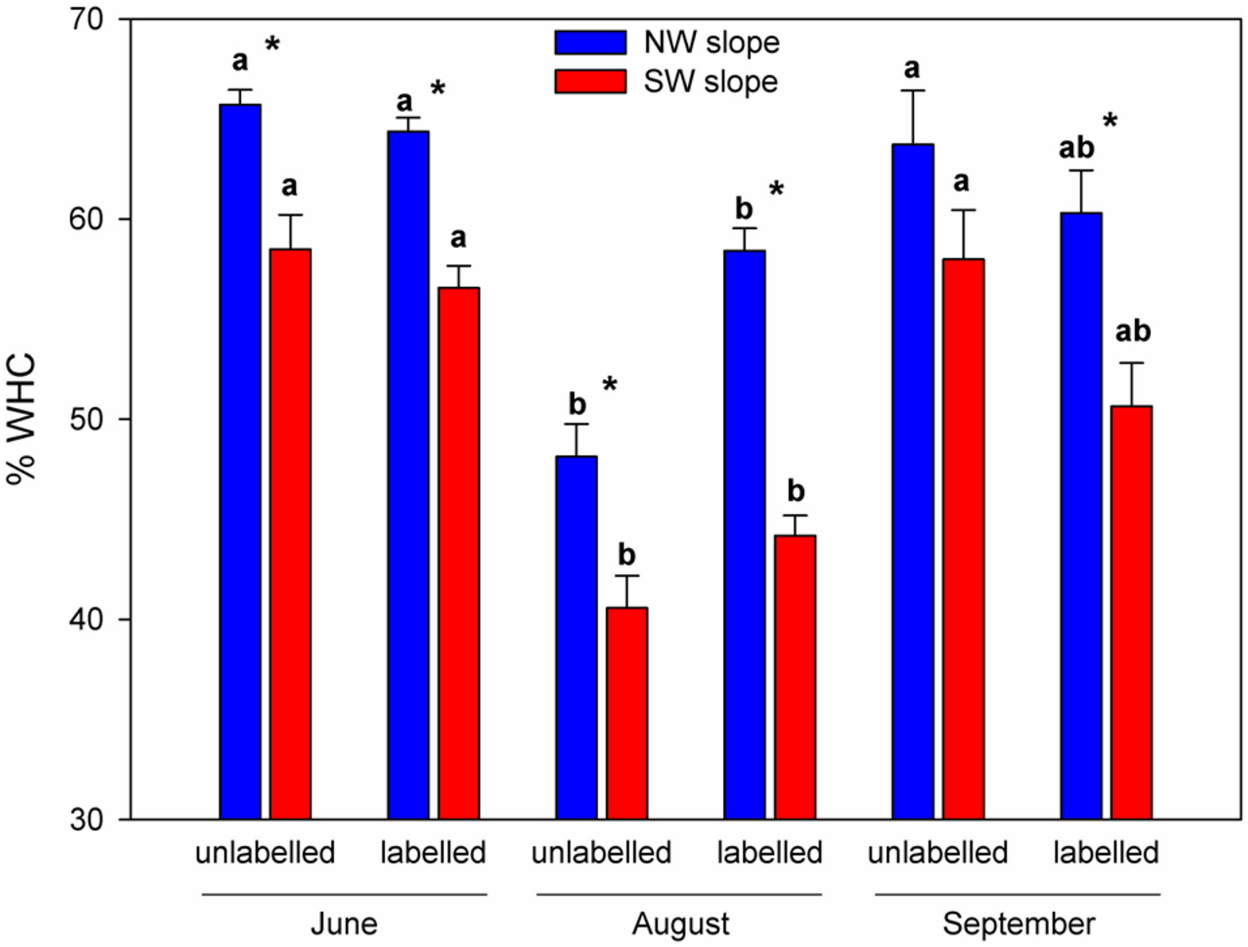
Gravimetric soil moisture related to water holding capacity (WHC) as determined from labelled (n = 48) and unlabelled (n = 4 to 8) beech-soil-mesocosms in June (ambient conditions at both exposures), August (intensified drought at SW exposure due to roof) and September (final harvest). Asterisks indicate significant differences (p<0.05) between NW and SW exposure at the respective harvest. Different indices indicate significant differences between different sampling dates and labelled and unlabelled beech-soil-mesocosms.

In order to evaluate whether N limitation could be a driver of the drought sensitivity of European beech, gross N turnover in intact beech seedling-soil-microbe-mesocosms was determined in the frame of a space-for-time translocation climate change experiment. Transfer from NW to SW increased soil temperature on average by 1°C (Fig 3) and—amplified by the roof—persistently decreased soil volumetric water content over the entire growing season (Fig 4). Also gravimetric water content was persistently lower in harvested mesocosms of the SW than of the NW treatments irrespective of addition of ^15^N label solution (Fig 5).

**Fig 6:**
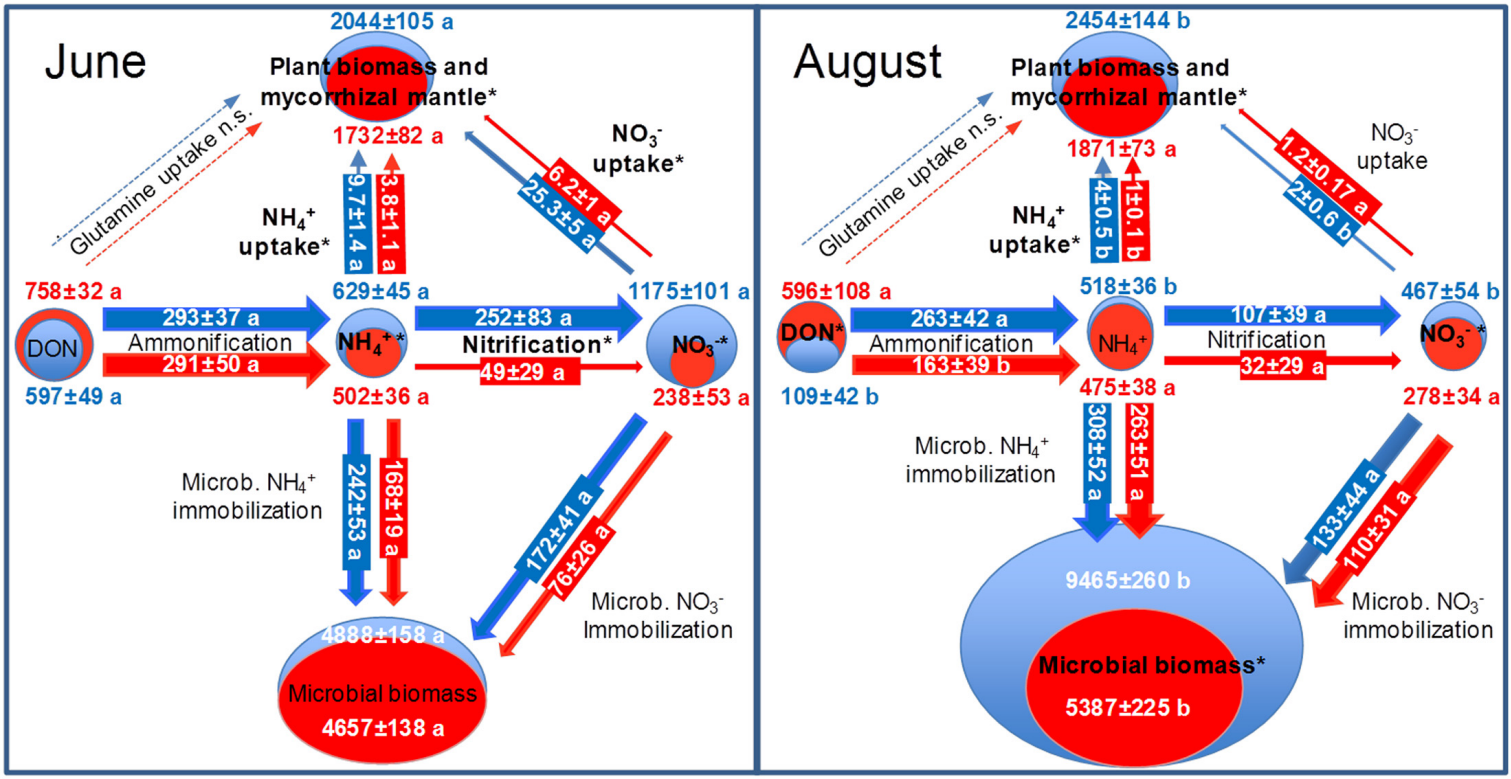
Gross N turnover rates (mg N m^-2^ day^-1^) (n = 8) and N pool sizes (mg N m^-2^) (N = 48) in intact beech seedling-soil-mesocosms. Blue: NW exposure (control treatment); Red: SW exposure (climate change treatment). The June sampling represents the onset of the growing season after full development of leaves, while the August sampling was conducted in the middle of the growing season after 39 days of rainfall exclusion at SW. Gross rates of N turnover were calculated based on ^15^N tracing and pool dilution approaches following homogeneous labelling of the intact soil with double-labelled ^15^N/^13^C-glutamine, ^15^NH_4_^+^, or ^15^NO_3_^-^. Thickness of process arrows and nitrogen pool signatures is representative for respective turnover rates and pool sizes. Processes and pools significantly affected by the climate change treatment are indicated by bold letters with asterisks. Different indices show significant differences between June and August for a given treatment/exposure.

Ammonification, nitrification and microbial immobilisation of inorganic N were approximately one order of magnitude larger than plant uptake (Fig 6). Microbial biomass N was several times larger than the plant N pool (Fig 6). Extractable soil NH_4_^+^ and NO_3_^-^ pools were in the magnitude of daily rates of gross inorganic N turnover. Nitrate was the dominant N source for beech seedlings in June, followed by NH_4_^+^, whereas organic N supplied as glutamine was hardly recovered (Fig 6). Plant N uptake was generally larger in June than in August (Fig 6).

Considering data from all sampling dates in our statistical model showed that transfer to SW increased the soil DON concentrations but decreased NH_4_^+^ availability, indicating impaired mineralization-immobilisation turnover with also reduced plant NH_4_^+^ uptake (Table 1, Fig 6). While the N cycle in soil of the NW control treatment was characterized by high nitrification rates, transfer to SW exposure resulted in a five-fold decline of gross nitrification and soil NO_3_^-^ concentrations already in June (Fig 6). Consequently, plant NO_3_^-^ uptake was also reduced by a factor of five (Fig 6), although mycorrhizal colonization of vital roots remained unaltered with values close to 100% in both exposures (Table 2).

**Table 1:** Results of two-way ANOVA analysis testing the factors exposure (NW vs. SW), time (June/August/September) and the interaction of exposure and time on gross rates of N turnover in the beech seedling-soil-microbe system and corresponding N pools.

	Exposure	Time	Exposure x Time
**Ammonification**	0.223	0.064	0.24
**Nitrification**	**0.010**	0.119	0.213
**Micr. NH**_**4**_^**+**^ **immob.**	0.223	0.084	0.733
**Micr. NO**_**3**_^**-**^ **immob.**	0.113	0.946	0.318
**Plant NH**_**4**_^**+**^ **uptake**	**<0.001**	0.002	0.633
**Plant NO**_**3**_^**-**^ **uptake**	**<0.001**	<0.001	0.002
**DON pool**	**<0.001**	<0.001	0.003
**NH**_**4**_^**+**^ **pool**	**0.034**	<0.001	0.506
**NO**_**3**_^**-**^ **pool**	**<0.001**	<0.001	<0.001
**Plant N pool**	**<0.001**	<0.001	0.517
**Micr. biomass N**	**0.0067**	<0.001	0.022

Numbers represent p values (significant at p<0.05). Significantly larger values in the control treatment are indicated by blue colour while red colour indicates larger values in the climate change treatment.

**Table 2:** Percentage of vital root tips colonized with mycorrhizal fungi.

	NW		SW		Two-Way-ANOVA		
	mean	SE	mean	SE		p	F
**June**	99.0	0.5	99.2	0.4	Slope	0.56	0.34
**August**	99.1	0.5	98.8	0.4	Time	0.08	2.51
**September**	99.7	0.1	99.9	0.1	Interactions	0.83	0.18

No significant differences in mycorrhizal colonization rates were observed between the control treatment (NW exposure) and the climate change treatment (SW exposure).

In conjunction with decreased NH_4_^+^ uptake, this resulted in a significantly diminished plant N pool (Fig 6). From June to August, plant biomass N increased significantly at NW but only marginally at SW under drought conditions (Fig 6). Plant N uptake calculated from short-term ^15^NH_4_^+^ and ^15^NO_3_^-^ tracing in June and August corresponded well with N uptake calculated from the plant N increment between June and August, with both approaches clearly showing a severely reduced uptake for SW (Table 3). In August, rates of N turnover and plant uptake were generally less than in June both at NW and SW with similar but less pronounced climate change treatment effects on N cycling as observed in August (Fig 6).

**Table 3:** Total plant N uptake (mg N m^-2^ day^-1^).

	N increment June-August (42 days) (mg N m^-2^ day^-1^)	Short-term ^15^NH_4_^+^ and ^15^NO_3_^-^ tracing June (mg N m^-2^ day^-1^)	Short-term ^15^NH_4_^+^ and ^15^NO_3_^-^ tracing August (mg N m^-2^ day^-1^)
**NW**	10	35 ± 7	6 ± 1.1
**SW**	3.4	9 ± 1.6	2.2 ± 0.31

Plant uptake was calculated (1) from the net increment of the mean plant N pool between June and August (n = 48 each), divided by the time span of 42 days between these sampling dates, (2) from the sum of NH4+ and NO3- uptake rates calculated from short-term (6h) 15NH4+ and 15NO3- tracing into plant biomass in June and August. Nitrogen uptake was always significantly smaller at SW exposure than at NW exposure (p<0.05).

**Fig 7:**
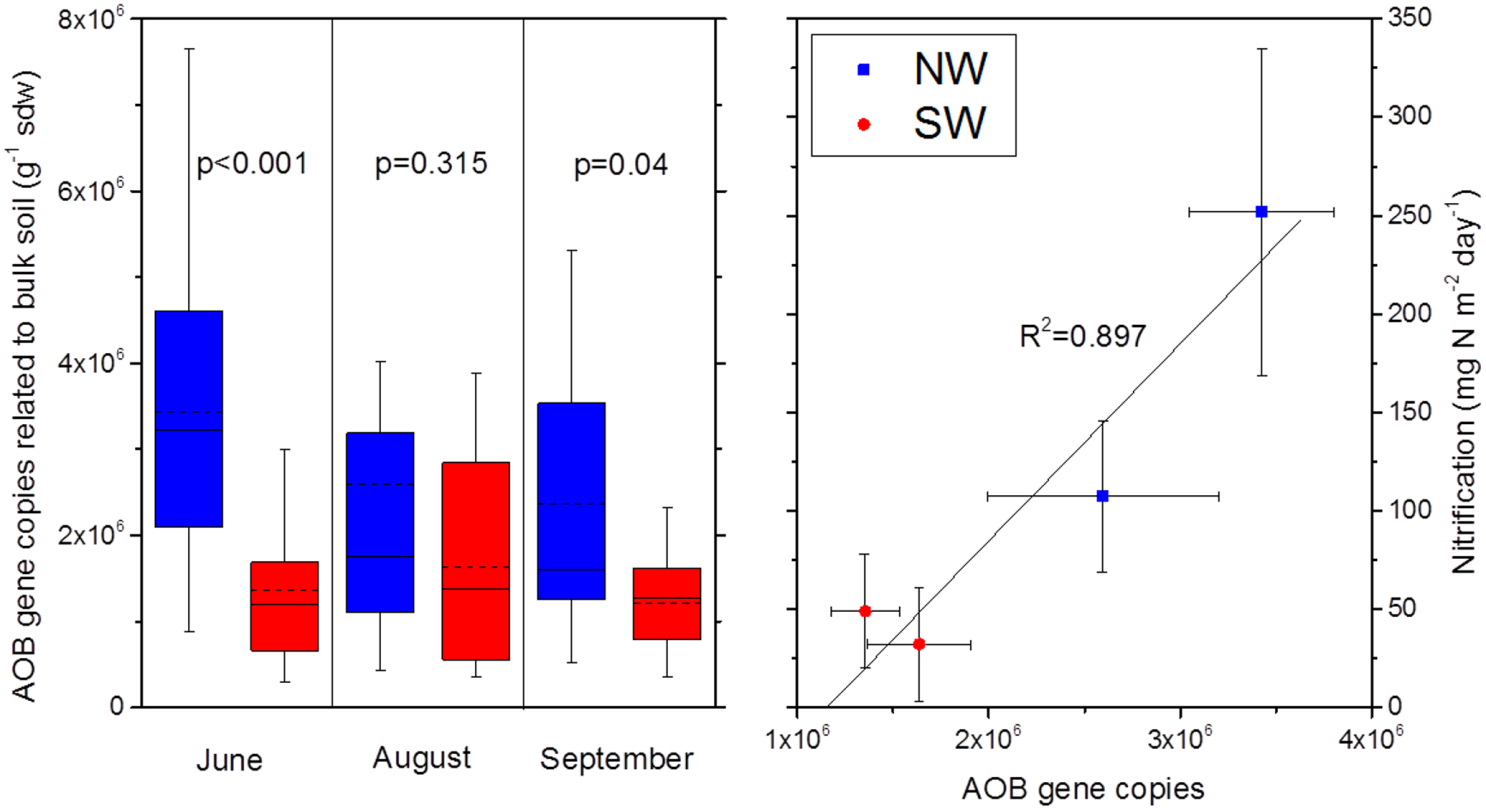
Ammonia oxidizing bacteria and nitrification rates. Abundance of genes encoding for ammonia monooxygenase of ammonia oxidizing bacteria in bulk soil (AOB, left panel) and relationships between AOB gene abundance and gross rates of nitrification (right panel). Blue colour: control treatment (NW exposure). Red colour: climate change treatment (SW exposure).

The observed effects of the climate change treatment on N processes (Table 1) in the plant-soil interface were confirmed by a range of supporting data. Concurrent analyses of microbial communities involved in selected processes of the N cycle revealed a pronounced reduction of ammonia oxidizing bacteria (AOB) in bulk soil in June and September and in the rhizosphere in August (Fig 7, S3 Table). Gross nitrification rates were strongly positively correlated with the abundance of AOB in soil (Fig 7).

**Fig 8:**
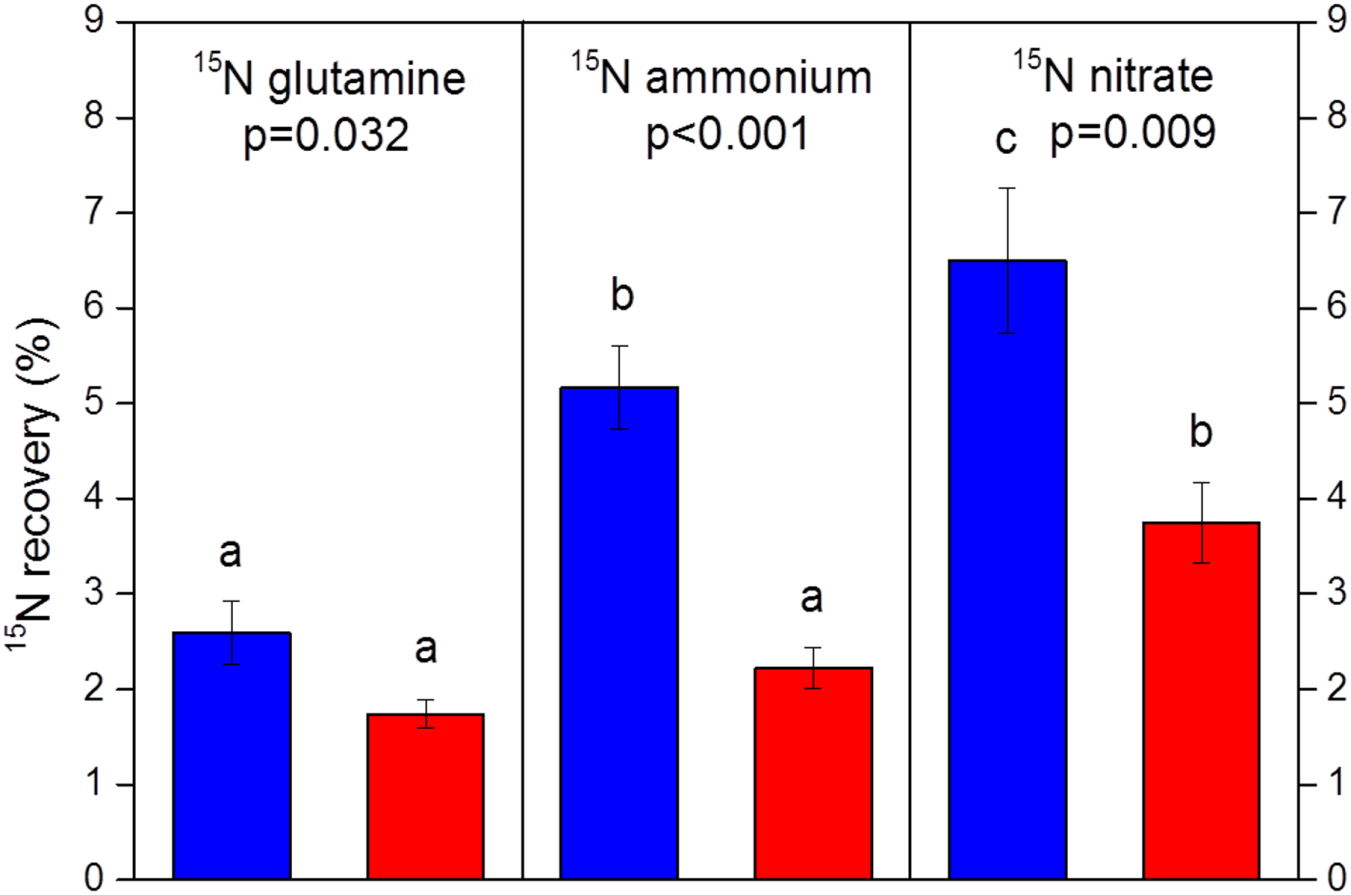
^15^N recovery (n = 8) in beech seedlings (sum of fine roots, coarse roots, stem and leaves). Data were collected in September, i.e., three months after isotope labelling with glutamine, NH_4_^+^ or NO_3_^-^ and indicate recovered % of isotopic excess, i.e., after subtracting ^15^N natural abundance. Blue: NW exposure (control treatment); red: SW exposure (climate change treatment). ^15^N recovery was highest after nitrate labelling both for SW and NW as indicated by different indices. The climate change treatment always reduced ^15^N recovery, as indicated by p<0.05.

**Fig 9:**
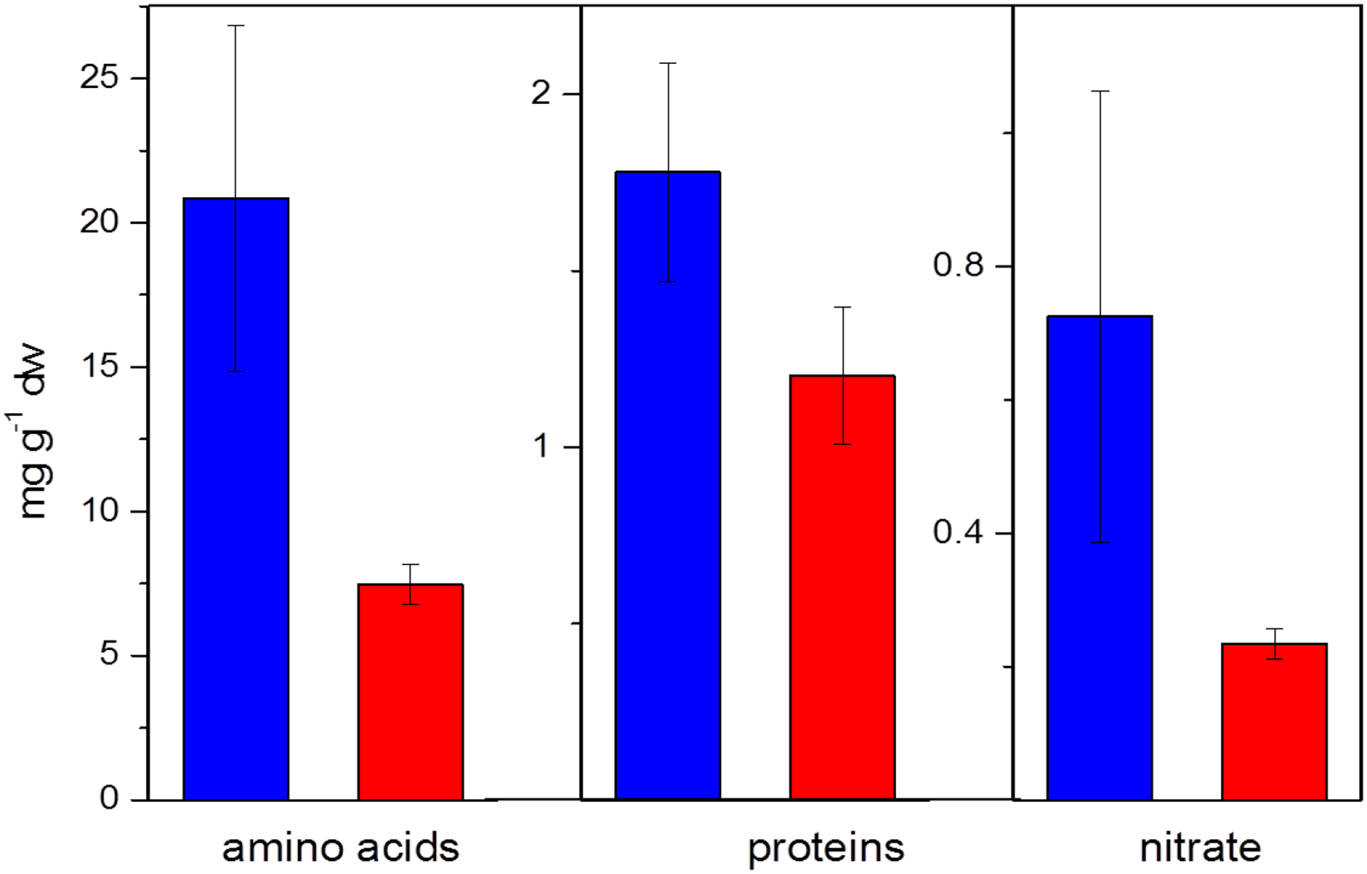
Metabolites (total amino acids, total soluble proteins, NO_3_^-^) extracted from fine roots of beech seedlings in June. Blue colour represents the control treatment (NW exposure), red colour represents the climate change treatment (SW exposure). Error bars denote standard errors of the mean (n = 4 per time and treatment). Amino acid and NO_3_^-^ metabolite levels were significantly lower in beech seedlings of the climate change treatment.

Reduced N uptake by beech seedlings of the climate change treatment is also supported by persistently higher ^15^N enrichment in mycorrhizal root tips grown in NW exposure than in SW exposure, irrespective of ^15^N labelled compound (S4 Table). Furthermore, ^15^N recovery in beech seedlings as determined in September, i.e., three months after ^15^N labelling was persistently smaller at SW than at NW regardless of the ^15^N source provided (Fig 8). Moreover, levels of N-bearing metabolites in fine roots of beech seedlings of the climate change treatment were reduced (Fig 9). Finally, above- and belowground biomass of beech seedlings were persistently smaller in SW than in NW beech-soil-mesocosms (Fig 6, S5 Table).

## Discussion

### Experimental and modelled climatic change

The statistical species range distribution modelling indicated a dramatic biome shift of European beech on calcareous soils, i.e., a reduction of almost 80% of the realized niche of beech in the year 2080. This approach is based on relationships to climate indicators and is—in contrast to process-based ecosystem models—neither based on physiological nor on biogeochemical processes. Furthermore, such predictions of the suitable growth area can underestimate the adaptive capacities of tree species [47]. Evidence for adaption of beech to drought conditions was observed close to their dry distribution limit [17].

Compared with the SRES A2 scenario used for the statistical modelling, the *in situ* space-for-time climate change experiment represents a smaller effect of climatic change. The NW exposure corresponds to a model climate for present day conditions of many beech forests in Central Europe, while the SW exposure is considered a model for climatic conditions expected in the target region for the following decades in the first half of the 21st century [48]. The differences in temperature across the valley of approx. 1°C correspond to expected climatic changes between the periods 1971–2000 and 2021–2050 [12,48]. Such a warming would correspond to an SRES B1 scenario, which has to be considered as very optimistic given the actual level of CO_2_ emissions [49]. We therefore used the A2 scenario as a framework for a potential development of the area of beech under climate change and rather difficult site conditions.

The fast and clear changes in N biogeochemistry and N nutrition observed here due to translocation from NW to SW exposure raise questions regarding the extent the beech stands in the investigated valley were affected by aspect in the past decades. Indeed these two beech stands—planted at both sides of the valley 80 years ago—are characterized by marked differences with 35% reduced basal area, 5 m less tree height and a timber volume of 236 rather than 349 solid m^3^ ha^-1^ at SW compared to NW exposure [48]. Hence, already the past microclimatic differences across the valley appeared to have been sufficient to impair the development of the beech stand at the warm-dry stand with SW exposure.

### Experimentally simulated climate change decelerates beech N nutrition as a consequence of impaired N cycling

Homogeneous and reproducible triple ^15^N isotope labelling allowed the simultaneous quantification of all major N turnover systems in the investigated intact beech seedling—soil-mesocosms under persistence of plant-microbe interactions during the experimental incubation period. This is expected to have resulted in comparable realistic rates of gross N turnover. The N cycle revealed by this approach was characterized by high rates of microbial mineralization–immobilisation turnover. The comparably low soil inorganic N concentrations in the range of daily rates of gross inorganic N production and consumption rates show that microbial N turnover largely controlled soil inorganic N availability. The dominance of NO_3_^-^ over NH_4_^+^ as a source for beech seedling N nutrition is in line with earlier studies at the site conducted with adult trees [15,16]. Generally significantly larger N uptake in June than in August (Fig 6) confirmed an earlier study [16], showing highest N uptake capacity of beech seedlings at the onset of the growing season. Such studies on N uptake capacity also indicated that glutamine is of high importance for N nutrition of beech seedlings [16,50,51]. Here, the homogeneous application of double-labelled ^13^C-carbon (^13^C)/^15^N glutamine to intact soil showed that ^15^N but not ^13^C was retrieved in mycorrhizal and plant tissues (Fig 6, S6 and S7 Tables). This suggests that either uptake of intact glutamine was not significant under such realistic field conditions or glutamine derived C was already subjected to respiration in the mycorrhizal mantle [18]. These findings show the limitation of uptake capacity studies to explain actual *in situ* uptake of organic N in the presence of microbial competition.

The most striking finding of the simulation of climatic change via transfer from NW to SW exposure was a chain of effects related to NO_3_^-^ production and consumption, i.e., the persistently reduced community of AOB and the associated five-fold decline of gross nitrification, soil NO_3_^-^ concentrations and beech NO_3_^-^ uptake. This suggests a tight link between soil water availability, nitrifier community, gross nitrification rates, soil NO_3_^-^ availability and N nutrition of beech seedlings. Apparently this microbial mediated effect of climate change on beech seedling N nutrition was strong enough to reduce growth and competitive performance of beech seedlings already after one year, as indicated by plant biomass and N content (Fig 6). A parallel study using the same experimental facilities as our work but focusing on the quantification of transcripts of functional N cycle genes found a persistent reduction of the transcripts of ammonia oxidizing archaea in the climate change treatment [52]. This indicates that—though not visible in the gene abundance levels—also the nitrifying activity of archaea was negatively affected by the exposure to simulated climate change, which probably also contributed to the observed reduction of gross nitrification rates.

The still significantly reduced number of gene copies of AOB at the end of the growing season in September in conjunction with the positive correlation of the abundance of AOB in soil and nitrification (Fig 7) suggest that the climate-change triggered mechanism of impaired nitrification activity with associated consequences for NO_3_^-^ availability and uptake by beech seedlings is persistent. This was also confirmed by the smaller long-term ^15^N recovery in beech seedlings in all labelling treatments (Fig 8) and the reduced N metabolite levels in the beech seedlings (Fig 9).

Ammonification and soil NH_4_^+^ levels were only marginally reduced in the climate change treatment (Fig 6). This was confirmed by a parallel study [52], which did not detect a significant reduction of transcript levels of the mineralization related genes chiA due to translocation from NW to SW exposure. Unaltered mineralization rates in the climate change treatment indicate that lower soil moisture accounted for attenuated nitrification through detraction of AOB metabolism and limitation of N substrate diffusion [53]. Soil moisture is the major environmental driver of *in situ* nitrification in Rendzic Leptosols with maximum nitrification at 65% of maximal water holding capacity [14], i.e., the soil water levels frequently found at NW exposure (Fig 5). A strong reduction of gross nitrification rates when soil moisture falls below this optimum is in line with our general understanding of nitrification [53] as well as with earlier observations for Rendzic Leptosols in beech stands [14]. The strong sensitivity of the AOB community to climatic change conditions may be related to the fact that these microorganisms obligatorily depend on ammonia oxidation without metabolic alternatives. Thus, NO_3_^−^-dominated N nutrition of beech seedlings may represent a major obstacle for beech performance under reduced soil water levels in marginal soil in a changing climate, leading to reduced growth and, thus, impaired competitive performance. The observed reduction of gross NO_3_^-^ supply at SW in June equals to 2 kg N ha^-1^ day^-1^ and thus has the potential to affect the N balance of the entire forest stand with its estimated N demand of roughly 100 kg N ha^-1^ growing season^-1^. Adult beech growing on Rendzic Leptosol has been observed to exhibit decreased maximum NO_3_^-^ uptake capacity and basal stem area with declining soil water availability [39] and has no opportunities to access deeper soil N pools in this shallow soil type.

It needs to be noted that the experimental findings from the space-for-time climate change experiment on the significant contribution of nutritional limitations to the drought sensitivity of European beech on marginal calcareous soil cannot be directly linked to the results of potential species range distribution modelling (Fig 2), as this statistical modelling approach is not based on physiology or biogeochemistry. The statistical model used in this study compares presence or absence of beech with climatic parameters only so that regional differences in N availability and their impacts on beech seedling N nutrition are not considered. The present study suggests that this problem could be tackled by the use of indices of N limitation such as atmospheric N deposition and soil C:N ratios in statistical species range models, which should improve their performance with regard to predicting regional differences in vulnerability and resilience of beech stands in a changing climate. Dynamic vegetation models so far focus on light and water as determinants of stand development and species competition in a changing climate. Our work shows that N dynamics is a critical component to be included in such models. The presented data facilitate testing whether dynamic vegetation models coupled to process-oriented biogeochemical ecosystem models [54] can consider effects of climate change on beech performance more comprehensively, taking also into account N availability as mediated by soil microbes. For this purpose, such biogeochemical test data on plant-soil-microbe N dynamics is urgently needed also for other forest stands. The inclusion of microbial nutrient dynamics in such models is also important in the light of recent observational evidence that nutrient availability could dominate as control of net ecosystem productivity in forests at a global scale [55].

### Implications for future forest management and ecosystem services

Impaired microbial provision of bioavailable N may be a stressor for beech in addition to plant physiological limitations under reduced soil water availability, enhancing the drought sensitivity of beech under predicted climatic changes. The nutritional deficiency could be further exacerbated by enhanced N leaching after prolonged drought due to retarded stabilization of microbial N in organo-mineral associations [40].

The results of the present study were gained for beech seedlings, however also could have direct implications for adult beech stands, because the shallow soils and the shallow root system do not allow adult beech to tap much deeper water and nutrient resources than beech seedlings. Furthermore, the competitive performance and establishment of beech seedlings is of importance for the rejuvenation of the stands. By highlighting the relationships between drought, N cycling and beech N seedling nutrition, our work provides pointers to developing mitigation options to increase beech seedling resilience and adaptation potential in a changing climate. In intensively managed forests and plantations, nutritional limitations could be counteracted by fertilization to increase levels of bioavailable N in soil. However, trade-offs such as undesired rapid hydrological NO_3_^-^ leaching in the joint aquifers of limestone karst systems would need to be quantified. The associated risk for nitrous oxide losses may remain small due to high nitrous oxide reductase activity in such soils, converting nitrous oxide into harmless dinitrogen [56]. Another option to promote beech seedling establishment and performance is silvicultural reduction of the stand density. This treatment increased soil water availability via increased throughfall and reduced competition for water in coniferous stands [57]. A further alternative is mixing beech stands with deep-rooting trees such as oak. Such mixing approaches appear to improve water balance of soil and beech via hydraulic lift of water from deeper aquifers followed by water efflux from oak roots [58], and thus may increase the resilience of beech seedlings to climate change in stands mixed with deep rooting tree species.

## Supporting Information

S1 Fig Supporting materials and methods. (DOCX)

S1 File Supporting materials and methods. (DOCX)

S1 Table Model statistics and accuracy measures for the fitted model. (DOCX)

S2 Table Primer sets and thermal profiles used for the absolute quantification of the respective genes. (DOCX)

S3 Table Abundance of microbes involved in nitrification based on the quantification of marker genes. (DOCX)

S4 Table ^15^N enrichment (atom%) in excess of natural abundance in mycorrhizal root tips. (DOCX)

S5 Table Aboveground and belowground dry plant biomass (mg) of beech seedlings for the three harvest dates. (DOCX)

S6 Table ^13^C recovery in plant. (DOCX)

S7 Table ^13^C enrichment (atom%) in excess of natural abundance in mycorrhizal root tips harvested in June, August and September. (DOCX)

## References

[1] Ellenberg H. (1996) Vegetation Mitteleuropas mit den Alpen, 5th edn (Ulmer, Stuttgart, Germany).

[2] Tarp P, Helles F, Holten-Andersen P, Larsen JB, Strange N (2000) Modelling near-natural silvicultural regimes for beech—an economic sensitivity analysis. Forest Ecology and Management 130: 187–198.

[3] Moosmayer H-U (2002) Langfristige regionale Waldbauplanung in Baden-Württemberg—Grundlagen und Ergebnisse. (Landesforstverwaltung Baden-Württemberg, Stuttgart, Germany).

[4] Geßler A, Keitel C, Kreuzwieser J, Matyssek R, Seiler W, Rennenberg H (2007) Potential risks for European beech (Fagus sylvatica L.) in a changing climate. Trees 21: 1–11.

[5] Rennenberg H, Dannenmann M, Gessler A, Kreuzwieser J, Simon J, Papen H (2009) Nitrogen balance in forest soils: nutritional limitation of plants under climate change stresses. Plant Biology 11, 4–23. 10.1111/j.1438-8677.2009.00241.x 19778364

[6] Kreuzwieser J, Geßler A (2010) Global climate change and tree nutrition: influence of water availability. Tree Physiology 30: 1221–1234. 10.1093/treephys/tpq055 20581013

[7] Leuschner C, Backes K, Hertel D, Schipka F, Schmitt U, Terborg O, et al (2001) Drought responses at leaf, stem and fine root levels of competitive Fagus sylvatica L. and Quercus petraea (Matt.) Liebl. trees in dry and wet years. Forest Ecology and Management 149: 33–46.

[8] Rennenberg H, Loreto F, Polle A, Brilli F, Fares S, Beniwal RS, et al (2006) Physiological responses of forest trees to heat and drought. Plant Biology 8: 556–571. 16773557 10.1055/s-2006-924084

[9] Michelot A, Bréda N, Damesin C, Dufrene E (2012) Differing growth response to climatic variations and soil water deficits of Fagus sylvatica, Quercus petraea and Pinus sylvestris in a temperate forest. Forest Ecology and Management 265: 161–171.

[10] Zimmermann J, Hauck M, Dulamsuren C, Leuschner C (2015) Climate warming-related growth decline affects Fagus sylvatica, but not other broad-leaved tree species in Central European Mixed forests. Ecosystems, in press (online first)

[11] Coumou D, Robinson A, Rahmstorf S (2013). Global increase in record-breaking monthly-mean temperatures. Climatic Change 118: 771–782.

[12] Wagner S, Berg P, Schädler G, Kunstmann H (2013) High resolution regional climate model simulations for Germany: Part II—projected climate changes. Climate Dynamics 40: 415–427.

[13] Smiatek G, Kunstmann H, Knoche R, Marx A (2009). Precipitation and temperature statistics in high-resolution regional climate models: Evaluation for the European Alps. Journal of Geophysical Research 114: D19107.

[14] Dannenmann M, Gasche R, Ledebuhr A, Papen H (2006) Effects of forest management on soil N cycling in beech forests stocking on calcareous soils. Plant and Soil 287: 279–300.

[15] Dannenmann M, Simon J, Gasche R, et al (2009) Tree girdling provides insight on the role of labile carbon in nitrogen partitioning between soil microorganisms and adult European beech. Soil Biology & Biochemistry 41: 1622–1631.

[16] Simon J, Dannenmann M, Gasche R, Holst J, Mayer H, Papen H, et al (2011) Competition for nitrogen between adult European beech and its offspring is reduced by avoidance strategy. Forest Ecology and Management 262: 105–114.

[17] Weber P, Bugmann H, Pluess AR, Walthert L, Rigling A (2013) Drought response and changing mean sensitivity of European beech close to the dry distribution limit. Trees 27: 171–181.

[18] Näsholm T, Kielland K, Ganeteg U (2009) Uptake of organic nitrogen by plants. New Phytologist 182: 31–48. 10.1111/j.1469-8137.2008.02751.x 19210725

[19] Schimel JP, Bennett J (2004) Nitrogen mineralization: challenges of a changing paradigm. Ecology 85, 591–602

[20] Chapman SK, Lanagley JA, Hart SC, Koch GW (2006) Plants actively control nitrogen cycling: uncorking the microbial bottleneck. New Phytologist 169, 27–34. 16390416 10.1111/j.1469-8137.2005.01571.x

[21] Jackson LE, Burger M, Cavagnaro TR (2008) Root nitrogen transformations, and ecosystem services. Annual Review of Plant Biology 59: 341–363. 10.1146/annurev.arplant.59.032607.092932 18444903

[22] Kuzyakov Y, Xu X (2013) Competition between roots and microorganisms for nitrogen: mechanisms and ecological relevance. New Phytologist 198: 656–669. 10.1111/nph.12235 23521345

[23] Geßler A, Schneider S, von Sengbusch D, Weber P, Hanemann U, Huber C et al, (1998) Field and laboratory experiments on net uptake of nitrate and ammonium by the roots of spruce (Picea abies) and beech (Fagus sylvatica) trees. New Phytologist 138: 275–328. 10.1046/j.1469-8137.1998.00107.x 33863096

[24] Lipson DA, Monson RK (1998) Plant-microbe competition for soil amino acids in the alpine tundra: effects of freeze-thaw and dry-rewet events. Oecologia 113: 406–414. 28307826 10.1007/s004420050393

[25] Jaeger CH, Monson RK, Fisk MC, Schmidt SK (1999) Seasonal partitioning of nitrogen by plants and soil microorganisms in an alpine ecosystem. Ecology 80: 1883–1891.

[26] Schimel JP, Jackson LE, Firestone MK (1989) Spatial and temporal effects on plant-microbial competition for inorganic nitrogen in a california annual grassland. Soil Biology & Biochemistry 21: 1059–1066.

[27] Jackson LE, Schimel JP, Firestone MK (1989) Short-term partitioning of ammonium and nitrate between plants and microbes in an annual grassland. Soil Biology & Biochemistry 21: 409–415.

[28] Dell CJ, Rice CW (2005) Short-term competition for ammonium and nitrate in tallgrass prairie. Soil Science Society of America Journal 69: 371–377.

[29] Harrison KA, Bol R, Bardgett RD (2007) Preferences for different nitrogen forms by coexisting plant species and soil microbes: Ecology 88, 989–999. 17536714 10.1890/06-1018

[30] Harrison KA, Bol R, Bardgett RD (2008). Do plant species with different growth strategies vary in their ability to compete with soil microbes for chemical forms of nitrogen? Soil Biology & Biochemistry 40: 228–237.

[31] Nadelhoffer KJ, Downs MR, Fry B (1999). Sinks for 15N-enriched additions to an oak forest and a red pine plantation. Ecological Applications 9: 72–86.

[32] Zak DR, Groffman PM, Pregitzer KS, Christensen S, Tiedje JM (1990) The vernal dam: Plant-microbe competition for nitrogen in the northern hardwood forests. Ecology 71: 651–656.

[33] Hart SC, Firestone MK (1991) Forest-floor mineral-soil interactions in the internal nitrogen-cycle of an old-growth forest. Biogeochemistry 12: 103–127.

[34] Johnson DW (1992) Nitrogen retention in forest soils. Journal of Environmental Quality 21: 1–12.

[35] Rennenberg H, Stoermer H, Weber P, Daum M, Papen H (2001) Competition of spruce trees for substrates of microbial N2O-production and -emission in a forest ecosystem. Journal of Applied Botany 75: 101–106.

[36] Finzi AC, Berthrong ST 2005 The uptake of amino acids by microbes and trees in three cold-temperate forests. Ecology 86: 3345–3353.

[37] Burger M, Jackson LE (2004) Plant and microbial nitrogen use and turnover: Rapid conversion of nitrate to ammonium in soil with roots: Plant and Soil 266, 289–301.

[38] Nakicenovic N, Davidson O, Davis G, de Bries B, Fenhann J, Gaffin S et al, (2000) Special Report on Emissions Scenarios: a special report of Working Group III of the Intergovernmental Panel on Climate Change, ISBN 92-9169-113-5.

[39] Geßler A, Jung K, Gasche R, Papen H, Heidenfelder A, Borner E et al (2005) Climate and forest management influence nitrogen balance of European beech forests: microbial N transformations and inorganic N net uptake capacity of mycorrhizal roots. European Journal of Forest Research 124: 95–111.

[40] Bimüller C, Dannenmann M, Tejedor J, von Lützow M, Buegger F, Meier R et al (2014) Prolonged summer droughts retard soil N processing and stabilization in organo-mineral fractions. Soil Biology & Biochemistry 68: 241–251.

[41] Wu H, Dannenmann M, Fanselow N, Wolf B, Yao ZS, Wu X et al (2011) Feedback of grazing on gross rates of N mineralization and inorganic N partitioning in steppe soils of Inner Mongolia. Plant and Soil: 340, 127–139.

[42] Guo C, Simon J, Gasche R, Naumann PS, Bimüller C, Pena R et al (2013) Minor contribution of leaf litter to N nutrition of (Fagus sylvatica) seedlings in a mountainous beech forest of Southern Germany. Plant and Soil 369, 657–668.

[43] Pena R, Offermann C, Simon J, Naumann PS, Gessler A, Holst J et al (2010) Girdling affects ectomycorrhizal fungal (EMF) diversity and reveals functional differences in EMF community composition in a beech forest. Appl. Environ. Microbiol. 76, 1831–1841. 10.1128/AEM.01703-09 20097809 PMC2837996

[44] Winter H., Lohais G. & Heldt W. (1992) Pholem transport of amino acids in relation to their cytosolic leaves in barley leaves. Plant Phys. 99, 996–1004. 10.1104/pp.99.3.996 PMC1080575 16669030

[45] Liu X-P, Grams TEE, Matyssek R, Rennenberg H (2005) Effects of elevated p03 on C- N- and S-metabolites in the leaves of juveniles beech and spruce differ between trees grown in moleculture and mixed culture. Plant Phys Biochem 43:147–154 10.1016/j.plaphy.2005.01.010 15820662

[46] Hanewinkel M, Cullmann D, Michiels HG, Kändler G (2014) Converting probabilistic tree species range shift projections into meaningful classes for management. Journal of Environmental Management 134: 153–165. 10.1016/j.jenvman.2014.01.010 24486469

[47] Hanewinkel M, Cullmann D, Schelhaas MJ, Nabuurs GJ, Zimmermann NE (2013) Climate change may cause severe loss in the economic value of European forest land. Nature Climate Change 3: 204–207.

[48] Geßler A, Keitel C, Nahm M, Rennenberg H (2004) Water shortage affects the water and nitrogen balance in Central European Beech Forests. Plant Biology 6: 289–298. 15143437 10.1055/s-2004-820878

[49] IPCC, 2014: Climate Change 2014: Synthesis Report Contribution of Working Groups I, II and III to the Fifth Assessment Report of the Intergovernmental Panel on Climate Change [Core Writing Team, R.K. Pachauri and L.A. Meyer (eds.)]. IPCC, Geneva, Switzerland, 151 pp

[50] Simon J, Waldhecker P, Brüggemann N, Rennenberg H (2010) Competition for nitrogen sources between European beech (Fagus sylvatica) and sycamore maple (Acer pseudoplatanus) seedlings. Plant Biology 12: 453–458. 10.1111/j.1438-8677.2009.00225.x 20522181

[51] Stoelken G, Simon J, Ehlting B, Rennenberg H (2010) The presence of amino acids affects inorganic N uptake in non-mycorrhizal seedlings of European beech (Fagus sylvatica L.). Tree Physiology 30: 1118–1128. 10.1093/treephys/tpq050 20595637

[52] Gschwendtner S, Tejedor J, Bimüller C, Dannenmann M, Kögel-Knabner I, Schloter M (2014) Climate Change induces shifts in abundance and activity pattern of Bacteria and Archaea catalyzing major transformation steps in nitrogen turnover in soil from a Mid-European beech forest. PLoSONE 9: e116614. 10.1371/journal.pone.0114278 PMC4252137 25462589

[53] Norton J, Stark JM (2011). Regulation and measurement of nitrification in terrestrial systems In: Klotz M. G. (ed.): Methods in Enzymology: Research on nitrification and related processes 486: 343–368 (Elsevier Academic Press, Burlington). 10.1016/B978-0-12-381294-0.00015-8 21185443

[54] Haas E, Klatt S, Fröhlich A, Kraft P, Werner C, Kiese R et al (2013) LandscapeDNDC: a process model for simulation of biosphere-atmosphere-hydrosphere exchange processes at site and regional scale. Landscape Ecology 28: 615–636.

[55] Fernández-Martinez M, Vicca S, Janssens IA, Sardans J, Luyssaert S, Campioli M et al (2014) Nutrient availability as the key regulator of global forest carbon balance. Nature Climate Change 4: 471–476.

[56] Dannenmann M, Butterbach-Bahl K, Gasche R, Willibald G, Papen H (2008) Dinitrogen emissions and the N2:N2O emission ratio of a Rendzic Leptosol as influenced by pH and forest thinning. Soil Biology & Biochemistry 40: 2317–2323.

[57] Kohler M, Nägele G, Sohn S, Bauhus J (2010) Can drought tolerance of Norway spruce (Picea abies (L.) Karst.) be increased through thinning? European Journal of Forest Research 129: 1109–1118.

[58] Pretzsch H, Schütze G, Uhl E (2013) Resistance of European tree species to drought stress in mixed versus pure forests: evidence of stress release by inter-specific facilitation. Plant Biology 15: 483–495. 10.1111/j.1438-8677.2012.00670.x 23062025

