# Supplementary material for: Climate Change Impairs Nitrogen Cycling in European Beech Forests
Source: PLoS One. 2016 Jul 13;11(7):e0158823. doi: 10.1371/journal.pone.0158823 (PMC4943676; doi:10.1371/journal.pone.0158823)
Supplement: S1 Fig — (DOCX) [file pone.0158823.s001.docx]

**S1 Fig. Histogram of predicted probabilities stratified by observed presence/absence (upper left), observed prevalence versus mean predicted probability by probability classes (upper right), the receiver operating characteristic (lower left) and threshold dependent accuracy measures versus possible thresholds (lower right).**
