# Supplementary material for: Climate Change Impairs Nitrogen Cycling in European Beech Forests
Source: PLoS One. 2016 Jul 13;11(7):e0158823. doi: 10.1371/journal.pone.0158823 (PMC4943676; doi:10.1371/journal.pone.0158823)
Supplement: S1 File — (DOCX) [file pone.0158823.s002.docx]

**Climate Change Impairs Nitrogen Cycling in European Beech Forests**

**Short Title: Nitrogen cycling in intact beech-soil systems**

Michael Dannenmann^1, 2*^ Carolin Bimüller^3$^, Silvia Gschwendtner^4$^, Martin Leberecht^5$^, Javier Tejedor^1$^, Silvija Bilela^2^, Rainer Gasche^1^, Marc Hanewinkel^8, 9^_,_ Andri Baltensweiler^9^_,_ Ingrid Kögel-Knabner^3,6^, Andrea Polle^5^, Michael Schloter^4^, Judy Simon^2, 10^, Heinz Rennenberg^2, 7^

^1^Institute of Meteorology and Climate Research, Atmospheric Environmental Research (IMK-IFU), Karlsruhe Institute of Technology (KIT), Kreuzeckbahnstrasse 19, 82467 Garmisch-Partenkirchen, Germany

^2^Institute of Forest Sciences, Chair of Tree Physiology, University of Freiburg; Georges-Koehler-Allee 53/54, 79110 Freiburg, Germany

^3^Lehrstuhl für Bodenkunde, Department für Ökologie und Ökosystemmanagement, Wissenschaftszentrum Weihenstephan. Technische Universität München, 85350 Freising-Weihenstephan, Germany

^4^Environmental Genomics, Helmholtz Zentrum München, German Research Center for Environmental Health, Ingolstädter Landstr. 1, 85764 Neuherberg, Germany

^5^Forest Botany and Tree Physiology, Büsgen-Institute, Georg-August Universität Göttingen, Büsgenweg 2, 37077 Göttingen, Germany

^6^IAS-Institute for Advanced Study, Technische Universität München, Lichtenbergstraße 2a, D-85748 Garching, Germany

^7^King Saud University, PO Box 2454, Riyadh 11451, Saudi Arabia

^8^Chair of Forestry Economics and Forest Planning, University of Freiburg, 79110 Freiburg, Germany

^9^Research Unit Forest Resources and Management, Swiss Federal Research Institute WSL, Zuercherstrasse 111, CH-8903 Birmensdorf, Switzerland

^10^Present adress: Chair of Plant Physiology & Biochemistry, University of Konstanz, Universitätsstrasse 10, 78457 Konstanz, Germany.

*Corresponding author: E-mail: michael.dannenmann@kit.edu

^$^These authors contributed equally to this work

**Supporting materials and methods: “Analysis of distribution of beech forests on calcareous soils in Europe”**

The map (Figure 1) is an intersect of a species distribution model for European beech [1] based on maps of geology for Europe. For the species distribution model we used a large dataset of presence/absence information for European beech derived from the ’Data on Crown Condition of the systematic grid (16 x 16 km)’ (Level I) from the ’International Co-operative Programme on Assessment and Monitoring of Air Pollution Effects on Forests’ (ICPF) [2] as response variable. The dataset with overall more than 8000 plots contains 1097 presence values for Fagus sylvatica. This response was modelled using derivations of precipitation and temperature from the WorldClim database as described in reference 3. For the initial model we used nineteen bioclimatic variables included in the database and an additional calculated set of six bioclimatic variables consisting of the number of days per year with mean temperature above 5 degree Celsius, the yearly heat sum above 5 degree Celsius, mean temperature and precipitation sum in summer (defined as the months May to September) and annual and summer drought index according to reference 4. For the projection under future conditions, we used output from the global circulation model HADCM3 [5] driven by the SRES scenario A2 [6] until the year 2080, which was calibrated and statistically downscaled to 30-arc-second tiles using the WorldClim data for ’current’ conditions.

As a statistical model formulation we fitted a Generalized Linear Model (GLM) with logit link functions, (i.e. logistic regressions). We used second-order polynomials of the described bioclimatic variables on the link scales of the GLM and reduced the number of the predictors using the sum of the adjusted deviances as a statistical measure. We calibrated the model by a stepwise selection checking for changes in the Bayesian Information Criterion [7]. The final five bioclimatic variables that entered the model in linear and quadratic form were: yearly sum of degree days above 5°C, iso-thermality, drought index after O’Neill et al. [4] over the year, sum of precipitation in the warmest quarter of the year and the precipitation of the most humid month. As the threshold value for presence or absence we used Cohen’s Kappa [8].

S1 Figure shows the histograms of predicted probabilities (upper left), the observed prevalence plotted against the mean predicted probabilities by probability classes (upper right), the ROC curve (lower left) and the threshold-dependent accuracy measures (lower right) for the modelled species European beech (Fagus sylvatica). The histograms of the predicted probabilities show a maximum at p= 0.0 for absence and between 0.4 and 0.5 for presence. The predicted probabilities are well distributed along the diagonal (upper right panel) and both, area under curve (AUC) of the receiver operating characteristic with a value of 0.86 (lower left panel) and (following [9]) Cohen’s Kappa with a maximum value of 0.43 (lower right panel) indicate good performance of the model.

To validate the model we carried out a 10-fold cross -validation. Therefore, we randomly split the data into a training and an evaluation dataset [10] and used 75% of the data records for training and the remaining 25% for evaluation [11].

S1 Table shows some statistical indices of the 10-fold cross-validation. The adjusted deviance was calculated according to [10] the displayed threshold maximizes Cohen’s Kappa. Clearly, the model appears to be robust with the cross validation means being very close to the model values, the variation within cross validation results being low and the cross validation results displaying not a single outlier.

A detailed description of the modelling approach, the choice and selection of the predictors and the database of the model used for this investigation can be found in reference 1. For a general overview on species distribution models for major tree species in Europe see also reference 12, specifically in the supplement of this publication.

Using the described model we produced two maps depicting the potential distribution of European beech under current climate (1950-2000) and for scenario A2 until the year 2080 and intersected these maps with maps of the geology of Europe [13]. In these European maps with a scale of 1: 5'000'000 [13] we selected all pixels that were assigned to formations including calcareous, limestone or other basic substrates. For Germany where no detailed information on the substrate was displayed in the European map, we used the 1:1'000'000 map for the Geology of Germany [14]. For areas in Europe such as Poland, Estonia, Lithuania and Latvia that did not display information on Geological map of Europe as well, we examined whether European beech plays a significant role in the species distribution. As this was not the case for both time periods we excluded these parts of Europe from the analysis. The intersected maps were created using standard overlay functions.

**Supporting materials and methods: “Calculation of N pools, isotope recovery and gross rates of N turnover”**

Measurements of N pool size in soil and plant as well as isotopic information of plant, soil organic, inorganic, microbial and mycorrhizal N pools was used to calculate gross rates of N turnover and isotope tracer recovery.

**Calculation of soil dry weight**

$$sdw (g)=sfw*\frac{D}{W}$$

sdw : soil dry weight (g)

sfw : soil fresh weight in the mini lysimeter (g)

D/W : quotient between dry soil after drying at 105°C and field fresh wet soil

**Total extractable soil N pool sizes**

**Ammonium-N pool (^14+15^**$\boldsymbol{NH}_{\boldsymbol{4}}^{\boldsymbol{+}}$**) [µg N/g sdw]**

$$\left[ {NH}_{4}^{+} \right]= \left( \left[ conc{NH}_{4}^{+} \right]-BW \right)*\left( \frac{VK_{2}SO_{4}+sfw-sdw}{sdw} \right)$$

concNH_4_^+^ : concentration of NH_4_^+^ in soil extract (mg N l^-1^)

BW = blind value $conc{NH}_{4}^{+}$-N in K_2_SO_4_

$VK_{2}SO_{4}$= volume 0.5M K_2_SO_4_ (ml)

**Nitrate pool (^14+15^**$\boldsymbol{NO}_{\boldsymbol{3}}^{\boldsymbol{-}}$**) [µg N/g sdw]**

$$\left[ {NO}_{3}^{-} \right]= \left( \left[ {concNO}_{3}^{-} \right]-BW \right)*\left( \frac{VK_{2}SO_{4}+sfw-asdw}{asdw} \right)$$

**Dissolved organic N pool (^14+15^DON) [mg N/kg sdw] calculated as TN_b_-DIN**

$$\left[ 14+15DON \right]=\left( \left[ {TN}_{b}-DIN \right]-BW \right)*\left( \frac{VK_{2}SO_{4}+sfw-asdw}{asdw} \right)$$

TNb: total chemically bound N in soil extracts (mg l^-1^)

DIN: dissolved inorganic N in soil extracts (mg l^-1^)

**Microbial biomass nitrogen** **(MBN)** **[mg N kg^-1^ sdw]; (X)_fum_= data from chloroform fumigated soil**

$\left[ MBN \right]\boldsymbol{=}$ $\left( \left[ {TN}_{b} \right]-BW*\left( \frac{VK_{2}SO_{4}+sfw-asdw}{asdw} \right) \right)_{fum}-\left( \left[ {TN}_{b} \right]-BW*\left( \frac{VK_{2}SO_{4}+sfw-asdw}{asdw} \right) \right)$

**Plant pool (P) [mg N mesocosm^-1^]**

$$\left[ P \right]=\left( \left[ P \right] \right)_{stem}+\left( \left[ P \right] \right)_{leaves}+\left( \left[ P \right] \right)_{\begin{aligned} coarse \\ roots \end{aligned}}+\left( \left[ P \right] \right)_{\begin{aligned} fine \\ roots \end{aligned}}$$

$$\left[ P \right]=\frac{pdw*\%N}{100}$$

pdw=total dry weight of plant tissue (mg)

Information on soil and plant N pools was transferred to the unit mg N m^-2^, considering the dry mass of soil contained in a beech-soil-mesocosm and/or the surface area.

**^15^N excess amount**

$\mathbf{NH}_{\mathbf{4}}^{\mathbf{+}}$**,** $\mathbf{NO}_{\mathbf{3}}^{\mathbf{-}}$**, DON [mg N kg^-1^ sdw]**

[^*^X]$=\left[ X \right]*\left( \frac{\left[ {}^{\%}X \right]_{t}-\left( \left[ {}^{\%}X \right]_{t} \right)_{\begin{aligned} nat \\ abund \end{aligned}}}{100} \right)$

[^*^X]= ^15^N excess amount (mg N kg^-1^ sdw) with X = ${NH}_{4}^{+}$, ${NO}_{3}^{-}$ or DON

[X]: N concentration in pool X (mg N kg^-1^ sdw)

[^%^X]: Atom% ^15^N enrichment of pool X

Nat abund: natural abundance ^15^N atom% excess (unlabelled soil).

**^15^N excess amount in microbial biomass** **(MBN) [mg N kg^-1^ sdw]**

$$\left[ {}^{*}{MBN} \right]=\frac{\left( \left[ {}^{\%}{MBN} \right]*\left[ {TN}_{b} \right] \right)_{fum}-\left[ {}^{\%}{MBN} \right]*\left[ {TN}_{b} \right]}{\left( \left[ {TN}_{b} \right] \right)_{fum}-\left[ {TN}_{b} \right]}$$

**^15^N excess amount in plant biomass [mg N lysimeter^-1^]**

$$\left[ {}^{*}P \right]_{t}=\left( \left( \left[ P \right]_{t} \right)_{stem}*\left( \left[ {}^{\%}P \right]_{t}-\left( \left[ {}^{\%}P \right]_{t} \right)_{\begin{aligned} nat \\ abund \end{aligned}} \right)_{stem} \right)+\left( \left( \left[ P \right]_{t} \right)_{leaves}*\left( \left[ {}^{\%}P \right]_{t}-\left( \left[ {}^{\%}P \right]_{t} \right)_{\begin{aligned} nat \\ abund \end{aligned}} \right)_{leaves} \right)+\left( \left( \left[ P \right]_{t} \right)_{\begin{aligned} coarse \\ roots \end{aligned}}*\left( \left[ {}^{\%}P \right]_{t}-\left( \left[ {}^{\%}P \right]_{t} \right)_{\begin{aligned} nat \\ abund \end{aligned}} \right)_{\begin{aligned} coarse \\ roots \end{aligned}} \right)+\left( \left( \left[ P \right]_{t} \right)_{\begin{aligned} fine \\ roots \end{aligned}}*\left( \left[ {}^{\%}P \right]_{t}-\left( \left[ {}^{\%}P \right]_{t} \right)_{\begin{aligned} nat \\ abund \end{aligned}} \right)_{\begin{aligned} fine \\ roots \end{aligned}} \right)$$

The recovery of ^15^N excess added by labelling was calculated by dividing the ^15^N excess amount detected in the investigated pools by the original amount of ^15^N excess added through the labelling solution.

**Calculation of gross rates of N turnover**

Gross rates of ammonification and nitrification were calculated following the ^15^N pool dilution equations given by [15].

**Ammonification [mg N kg^-1^ sdw day^-1^]**

$$\boldsymbol{=}\frac{\left[ {NH}_{4}^{+} \right]_{6}-\left[ {NH}_{4}^{+} \right]_{48}}{1.75 days}*ln\left( \frac{\left[ {}^{*}{{NH}_{4}^{+}} \right]_{6}*\left[ {NH}_{4}^{+} \right]_{48}}{\left[ {}^{*}{{NH}_{4}^{+}} \right]_{48}*\left[ {NH}_{4}^{+} \right]_{6}} \right)\% ln\frac{\left[ {NH}_{4}^{+} \right]_{6}}{\left[ {NH}_{4}^{+} \right]_{48}}$$

Subscript [ ]: Measurement time (0 = before labelling; 6 = 6 hours after ^15^N application; 48 = 48 hours after ^15^N application)

**Nitrification [mg N kg^-1^ sdw day^-1^]**

$$\boldsymbol{=}\frac{\left[ {NO}_{3}^{-} \right]_{6}-\left[ {NO}_{3}^{-} \right]_{48}}{1.75 days}*ln\left( \frac{\left[ {}^{*}{{NO}_{3}^{-}} \right]_{6}*\left[ {NO}_{3}^{-} \right]_{48}}{\left[ {}^{*}{{NO}_{3}^{-}} \right]_{48}*\left[ {NO}_{3}^{-} \right]_{6}} \right)\% ln\frac{\left[ {NO}_{3}^{-} \right]_{6}}{\left[ {NO}_{3}^{-} \right]_{48}}$$

Plant N uptake and microbial immobilization of inorganic N was calculated based on short-term tracing (6 hours) of labelled substances (^15^NH_4_^+^, ^15^NO_3_^-^) into plant biomass. The short period was chosen to minimize bias due to unquantified tracer outflow from the sink pools and depletion of tracer in the source pool [16, 17].

**Plant** $\mathbf{NH}_{\mathbf{4}}^{\mathbf{+}}$ **uptake [mg N lysimeter^-1^ day^-1^]**

$$\boldsymbol{=}\frac{\left[ {}^{*}P \right]_{6}}{Average\left( \left[ {}^{APE}{{NH}_{4}^{+}} \right]_{0} \right.;\left. \left[ {}^{APE}{{NH}_{4}^{+}} \right]_{6} \right)*0.25 days}$$

[APE]: Atom% excess of pool X

$\left[ {}^{APE}{{NH}_{4}^{+}} \right]_{0}$= ${}^{15}{{NH}_{4}^{+}}$- N atomic % excess immediately after ^15^N labelling

$\left[ {}^{APE}{{NH}_{4}^{+}} \right]_{6}={}^{15}{{NH}_{4}^{+}}$- N atomic % excess six hours after labelling.

**Plant** $\mathbf{NO}_{\mathbf{3}}^{\mathbf{-}}$ **uptake [mg N lysimeter^-1^]**

$$\boldsymbol{=}\frac{\left[ {}^{*}P \right]_{6}}{Average\left( \left[ {}^{APE}{{NO}_{3}^{-}} \right]_{0} \right.;\left. \left[ {}^{APE}{{NO}_{3}^{-}} \right]_{6} \right)*0.25 days}$$

**Microbial** $\mathbf{NH}_{\mathbf{4}}^{\mathbf{+}}$ **inmobilization [mg N kg^-1^ sdw day^-1^]**

$$\boldsymbol{=}\frac{\left[ MB \right]_{6}*\left[ {}^{*}{MB} \right]_{6}}{Average\left( \left[ {}^{APE}{{NH}_{4}^{+}} \right]_{0} \right.;\left. \left[ {}^{APE}{{NH}_{4}^{+}} \right]_{6} \right)*0.25 days}$$

**Microbial** $\mathbf{NO}_{\mathbf{3}}^{\mathbf{-}}$ **immobilization [mg N kg^-1^ sdw day^-1^].**

$$\boldsymbol{=}\frac{\left[ MB \right]_{6}*\left[ {}^{*}{MB} \right]_{6}}{Average\left( \left[ {}^{APE}{{NO}_{3}^{-}} \right]_{0} \right.;\left. \left[ {}^{APE}{{NO}_{3}^{-}} \right]_{6} \right)*0.25 days}$$

All N turnover rates were transferred to the unit mg N m^-2^ day^-1^, considering total dry soil contained in the beech-soil-mesocosm and the surface area. Plant uptake rates were transferred to the same unit by considering the surface area of the beech-soil-mesocosm.

**References**

1. Hanewinkel, M., Cullmann, D., Michiels, H.G. & Kändler, G. Converting probabilistic tree species range shift projections into meaningful classes for management. *J. Env. Manage.* **134,** 153-165 (2014).
2. Fischer, R., et al. The Condition of Forests in Europe. 2010 Executive Report (ICP Forests and European Commission, Hamburg and Brussels, 21 pp., 2010).
3. Hijmans, R., S., Cameron, E., Parra, J., Jones, P. & Jarvis, A. 2005. Very high resolution interpolated climate surfaces for global land areas. *Int. J. Clim.* **25**, 1965-1978 (2005).
4. O'Neill, G. A., Nigh, G., Wang, T. & Ott, P. K. Growth response functions improved by accounting for nonclimatic site effects. *Can. J. For. Res.* **37**, 2724-2730 (2007)
5. Gordon, C., et al. The simulation of sst, sea ice extents and ocean heat transports in a version of the hadley centre coupled model without flux adjustments. *Clim. Dyn.* **16**, 147-168 (2000).
6. Nakicenovic, N., et al. Special Report on Emissions Scenarios: a special report of Working Group III of the Intergovernmental Panel on Climate Change, ISBN 92-9169-113-5, (2000).
7. Schwarz, G. Estimating the Dimension of a Model. *Annals of Statistics* *6*, 461-464 (1978).
8. Cohen, J. A coefficient of agreement for nominal scales. *Educational and Psychological Measurement* **24**, 37-46 (1960).
9. Landis, J. R. and G. G. Koch. The measurement of observer agreement for categorical data. Biometrics 33:159-174 (1977).
10. Guisan, A. and N. E. Zimmermann. Predictive habitat distribution models in ecology. Ecological Modelling 135:147-186 (2000).
11. Fielding, A. H. and J. F. Bell. A review of methods for the assessment of prediction errors in conservation presence/absence models. Environmental Conservation 24:38-49 (1997).
12. Hanewinkel, M., Cullmann, D., Schelhaas, M. J., Nabuurs, G. J. & Zimmermann, N. E. Climate change may cause severe loss in the economic value of European forest land. *Nature Clim. Ch.* **3**, 204-207 (2013).
13. Asch, K. [The 1 : 5 Million International Geological Map of Europe and Adjacent Areas](http://www.geoshop-hannover.de/is-bin/INTERSHOP.enfinity/WFS/port_bgr/de_DE/-/EUR/OG_ViewProductDetails-View?ProductRef=bgr_188010003%40port_bgr&OG_MainIDs=20193&PageID=0&Avalability=true&CategoryID=Geologie&SubcategoryID=PUB-0010_0) (German Federal Agency for Geosciences and Raw Materials BGR, Hannover, 2003). <http://www.bgr.bund.de/EN/Themen/SammlungenGundlagen/GG_geol_Info/Europa/IGME5000/igme5000_inhalt_en.html?nn=1556480>
14. German Federal Agency for Geosciences and Raw Materials BGR. Geological Map of Germany, Scale 1:1000000, 4th edition. (BGR, Hannover 1993). <http://www.bgr.bund.de/EN/Themen/Sammlungen-Grundlagen/GG_geol_Info/Karten/Deutschland/GK1000/gk1000_inhalt_en.html>
15. Kirkham, D. & Bartholomew, W. V. Equations for following nutrient transformations in soil utilizing tracer data. *Soil Sci. Soc. Am. Proc.* **18**, 33-34 (1954).
16. Wu, H., et al. Feedback of grazing on gross rates of N mineralization and inorganic N partitioning in steppe soils of Inner Mongolia. *Plant Soil*, **340**, 127–139 (2011)
17. Stark, J.M. [Nutrient Transformations.](http://www.biology.usu.edu/files/uploads/Faculty/Stark-J/Stark2000-Methods%20in%20Ecosystem%20Science.pdf) Pages 215-234 in O.E. Sala, R.B. Jackson, H.A. Mooney, and R. Howarth (eds.), Methods in Ecosystem Science (Springer, New York, 2000).
