# Supplementary material for: Climate Change Impairs Nitrogen Cycling in European Beech Forests
Source: PLoS One. 2016 Jul 13;11(7):e0158823. doi: 10.1371/journal.pone.0158823 (PMC4943676; doi:10.1371/journal.pone.0158823)
Supplement: S1 Table — (DOCX) [file pone.0158823.s003.docx]

**S1 Table. Model statistics and accuracy measures for the fitted model, mean (Mean CV) of the 10-fold cross-validation statistics and measures for the 10-fold cross-validation (CV1-CV10). CK CI-low and CK CI-up denote the lower and upper bound of the 0.95-confidence interval for Cohen's kappa, respectively.**

|  | CV 1 | CV 2 | CV 3 | CV 4 | CV 5 | CV 6 | CV 7 | CV 8 | CV 9 | CV10 | Mean |
| --- | --- | --- | --- | --- | --- | --- | --- | --- | --- | --- | --- |
| deviance | 0.281 | 0.281 | 0.281 | 0.283 | 0.286 | 0.276 | 0.289 | 0.277 | 0.275 | 0.280 | 0.281 |
| Adj. deviance | 0.280 | 0.280 | 0.280 | 0.283 | 0.285 | 0.276 | 0.289 | 0.276 | 0.274 | 0.279 | 0.280 |
| Threshold | 0.270 | 0.270 | 0.270 | 0.260 | 0.265 | 0.260 | 0.270 | 0.265 | 0.265 | 0.270 | 0.266 |
| AUC | 0.864 | 0.853 | 0.862 | 0.851 | 0.839 | 0.879 | 0.820 | 0.885 | 0.891 | 0.872 | 0.861 |
| Cohen´s Kappa | 0.433 | 0.434 | 0.429 | 0.434 | 0.431 | 0.424 | 0.436 | 0.427 | 0.422 | 0.433 | 0.430 |
| CK Cl-low | 0.402 | 0.402 | 0.398 | 0.402 | 0.400 | 0.392 | 0.405 | 0.395 | 0.391 | 0.401 | 0.399 |
| CK Cl-up | 0.465 | 0.465 | 0.460 | 0.465 | 0.463 | 0.455 | 0.467 | 0.458 | 0.454 | 0.464 | 0.462 |
