# Supplementary material for: Climate Change Impairs Nitrogen Cycling in European Beech Forests
Source: PLoS One. 2016 Jul 13;11(7):e0158823. doi: 10.1371/journal.pone.0158823 (PMC4943676; doi:10.1371/journal.pone.0158823)
Supplement: S2 Table — (DOCX) [file pone.0158823.s004.docx]

**S2 Table. Primer sets and thermal profiles used for the absolute quantification of the respective genes**

| **Target gene** | **Source of standard** | **Primer** | **References** | **Thermal profile** | **No. of cycles** |
| --- | --- | --- | --- | --- | --- |
| AOA | *Nitrosomonas europaea* | amo19F, CrenamoA16r48x | 28,29 | 94°C-45s/ 55°C-45s/ 72°C-45s | 40 |
| AOB | *Fosmid clone 54d9* | amoA1F, amoA2R | 30 | 94°C-45s/ 59°C-45s/ 72°C-45s | 40 |
|  |  |  |  |  |  |

* Touchdown: -1°C per cycle
