# Supplementary material for: Climate Change Impairs Nitrogen Cycling in European Beech Forests
Source: PLoS One. 2016 Jul 13;11(7):e0158823. doi: 10.1371/journal.pone.0158823 (PMC4943676; doi:10.1371/journal.pone.0158823)
Supplement: S3 Table — (DOCX) [file pone.0158823.s005.docx]

**S3 Table. Abundance of microbes involved in nitrification based on the quantification of marker genes.**

|  |  | **Bulk soil** | | | | **Rhizosphere** | | | |
| --- | --- | --- | --- | --- | --- | --- | --- | --- | --- |
|  |  | **NW** | SD | **SW** | SD | **NW** | SD | **SW** | SD |
|  | June | 1.5x10^8^ | 1.4x10^8^ | 1.2x10^8^ | 1.1x10^8^ | 6.1x10^8^ | 6.9x10^8^ | 8.9x10^8^ | 8.9x10^8^ |
| **AOA** | August | 1.3x10^8^ | 1.5x10^8^ | 1.5x10^8^ | 1.8x10^8^ | 9.8x10^8^ | 5.3x10^8^ | 1.0x10^9^ | 1.1x10^9^ |
|  | September | 5.8x10^7^ | 4.0x10^7^ | 2.5x10^8^ | 2.6x10^8^ | 2.1x10^8^ | 1.1x10^8^ | 5.4x10^8^ | 6.3x10^8^ |
|  | June | 3.4x10^6^ | 1.8x10^6^ | 1.4x10^6^ | 8.7x10^5^ | 3.0x10^7^ | 2.3x10^7^ | 1.9x10^7^ | 1.4x10^7^ |
| **AOB** | August | 2.6x10^6^ | 2.7x10^6^ | 1.6x10^6^ | 1.1x10^6^ | 2.7x10^7^ | 1.1x10^7^ | 1.4x10^7^ | 1.7x10^7^ |
|  | September | 2.4x10^6^ | 1.6x10^6^ | 1.2x10^6^ | 5.8x10^5^ | 6.4x10^6^ | 2.4x10^6^ | 9.3x10^6^ | 9.4x10^6^ |

Archaeal and bacterial nitrifiers based on the ammonia monooxygenase gene amoA. Significantly more gene copies in soil at NW exposure compared to SW exposure are highlighted in dark blue, while dark red is indicating significantly larger values at SW exposure. SD: standard deviation of the mean.
