# Supplementary material for: Climate Change Impairs Nitrogen Cycling in European Beech Forests
Source: PLoS One. 2016 Jul 13;11(7):e0158823. doi: 10.1371/journal.pone.0158823 (PMC4943676; doi:10.1371/journal.pone.0158823)
Supplement: S4 Table — (DOCX) [file pone.0158823.s006.docx]

**S4 Table. ^15^N enrichment (atom%) in excess of natural abundance in mycorrhizal root tips harvested in June and August (6 and 48 hours after isotope labelling each) and in September (3 months after isotope labelling).**

|  | NW | | SW | | Two-Way-ANOVA | | |
| --- | --- | --- | --- | --- | --- | --- | --- |
| N form | mean | SE | mean | SE |  | p | F |
| **Glutamine** |  |  |  |  |  |  |  |
| **June_6h** | 0.0166 | 0.0022 | 0.0018 | 0.0003 | Exposure | 0.000 | 258 |
| **June_48h** | 0.0575 | 0.0166 | 0.0048 | 0.0030 | Time | 0.000 | 274.9 |
| **August_6h** | 0.0266 | 0.0113 | 0.0052 | 0.0014 | Interactions | 0.000 | 147.1 |
| **August_48h** | 0.0491 | 0.0157 | 0.0259 | 0.0111 |  |  |  |
| **September_3 months** | 0.0690 | 0.0076 | 0.0610 | 0.0085 |  |  |  |
| **Ammonium** |  |  |  |  |  |  |  |
| **June_6h** | 0.1586 | 0.0432 | 0.2157 | 0.0634 | Exposure | 0.049 | 4.1 |
| **June_48h** | 0.4825 | 0.0847 | 0.3964 | 0.0624 | Time | 0.000 | 11.6 |
| **August_6h** | 0.2371 | 0.0668 | 0.0833 | 0.0437 | Interactions | 0.534 | 0.8 |
| **August_48h** | 0.5961 | 0.1149 | 0.4223 | 0.1737 |  |  |  |
| **September_3 months** | 0.7681 | 0.1160 | 0.5523 | 0.0894 |  |  |  |
| **Nitrate** |  |  |  |  |  |  |  |
| **June_6h** | 0.0648 | 0.0252 | 0.0551 | 0.0136 | Exposure | 0.005 | 8.8 |
| **June_48h** | 0.2155 | 0.0546 | 0.1027 | 0.0327 | Time | 0.010 | 3.7 |
| **August_6h** | 0.0960 | 0.0264 | 0.0339 | 0.0085 | Interactions | 0.269 | 1.3 |
| **August_48h** | 0.2387 | 0.0790 | 0.0621 | 0.0161 |  |  |  |
| **September_3 months** | 0.1871 | 0.0445 | 0.1615 | 0.0448 |  |  |  |

Significantly higher ^15^N enrichments at NW compared to SW is indicated by dark blue colour.

|  |  | **June** | **August** | **September** |
| --- | --- | --- | --- | --- |
| **Aboveground** | NW | 2325±134 | 2590±155 | 1919±93 |
|  | SW | 2031±106 | 2065±90 | 1762±98 |
| **Belowground** | NW | 1893±98 | 2421±155 | 2789±126 |
|  | SW | 1546±7 | 1576±60 | 1955±96 |

**Table S5: Aboveground and belowground dry plant biomass (mg) of beech seedlings for the three harvest dates. n=48 (June and August); n=24 (September). Significantly larger biomass at NW compared to SW tested for single harvest dates is indicated by dark blue colour.**

|  | **Mean**  **[mg ^13^C excess]** | **SD** | **% of applied**  **^13^C excess** | **Mean**  **[mg ^13^C excess]** | **SD** |
| --- | --- | --- | --- | --- | --- |
| **June_6h** | 0.0072 | 0.0078 | 0.4280 | -0.0028 | 0.0031 |
| **June_48h** | -0.0006 | 0.0032 | -0.0347 | -0.0127 | 0.0082 |
| **August_6h** | 0.0003 | 0.0056 | 0.0194 | -0.0007 | 0.0031 |
| **August_48h** | 0.0002 | 0.0048 | 0.0108 | 0.0005 | 0.0043 |
| **September_3months** | 0.0248 | 0.0086 | 1.4719 | 0.0117 | 0.0112 |

**Table S6: ^13^C recovery (mg ^13^C excess, and % of ^13^C excess applied via glutamine) in plant (sum of fine roots, coarse roots, stem, leaves) for the single harvesting dates (month_time after glutamine labelling). No significant differences were observed between NW and SW.**

| **Harvest date_time after ^13^C labelling** | NW | | SW | | Two-Way-ANOVA | | |
| --- | --- | --- | --- | --- | --- | --- | --- |
|  | mean | SE | mean | SE |  | p | F |
| **June_6h** | -0.0004 | 0.0001 | -0.0006 | 0.0003 | Exposure | 0.924 | 0.009 |
| **June_48h** | -0.0002 | 0.0002 | -0.0014 | 0.0003 | Time | **0.000** | 6.178 |
| **August_6h** | 0.0005 | 0.0006 | 0.0000 | 0.0005 | Interactions | **0.003** | 4.602 |
| **August_48h** | -0.0010 | 0.0003 | -0.0007 | 0.0004 |  |  |  |
| **September_3 months** | -0.0003 | 0.0005 | 0.0012 | 0.0002 |  |  |  |

**Table S7: ^13^C enrichment (atom%) in excess of natural abundance in mycorrhizal root tips harvested in June, August and September. No significant ^13^C excess enrichment was observed in mycorrhiza within 48 hours after ^13^C glutamine labelling.**

**References**

1. Hanewinkel, M., Cullmann, D., Michiels, H.G. & Kändler, G. Converting probabilistic tree species range shift projections into meaningful classes for management. *J. Env. Manage.* **134,** 153-165 (2014).
2. Fischer, R., et al. The Condition of Forests in Europe. 2010 Executive Report (ICP Forests and European Commission, Hamburg and Brussels, 21 pp., 2010).
3. Hijmans, R., S., Cameron, E., Parra, J., Jones, P. & Jarvis, A. 2005. Very high resolution interpolated climate surfaces for global land areas. *Int. J. Clim.* **25**, 1965-1978 (2005).
4. O'Neill, G. A., Nigh, G., Wang, T. & Ott, P. K. Growth response functions improved by accounting for nonclimatic site effects. *Can. J. For. Res.* **37**, 2724-2730 (2007)
5. Gordon, C., et al. The simulation of sst, sea ice extents and ocean heat transports in a version of the hadley centre coupled model without flux adjustments. *Clim. Dyn.* **16**, 147-168 (2000).
6. Nakicenovic, N., et al. Special Report on Emissions Scenarios: a special report of Working Group III of the Intergovernmental Panel on Climate Change, ISBN 92-9169-113-5, (2000).
7. Schwarz, G. Estimating the Dimension of a Model. *Annals of Statistics* *6*, 461-464 (1978).
8. Cohen, J. A coefficient of agreement for nominal scales. *Educational and Psychological Measurement* **24**, 37-46 (1960).
9. Landis, J. R. and G. G. Koch. The measurement of observer agreement for categorical data. Biometrics 33:159-174 (1977).
10. Guisan, A. and N. E. Zimmermann. Predictive habitat distribution models in ecology. Ecological Modelling 135:147-186 (2000).
11. Fielding, A. H. and J. F. Bell. A review of methods for the assessment of prediction errors in conservation presence/absence models. Environmental Conservation 24:38-49 (1997).
12. Hanewinkel, M., Cullmann, D., Schelhaas, M. J., Nabuurs, G. J. & Zimmermann, N. E. Climate change may cause severe loss in the economic value of European forest land. *Nature Clim. Ch.* **3**, 204-207 (2013).
13. Asch, K. [The 1 : 5 Million International Geological Map of Europe and Adjacent Areas](http://www.geoshop-hannover.de/is-bin/INTERSHOP.enfinity/WFS/port_bgr/de_DE/-/EUR/OG_ViewProductDetails-View?ProductRef=bgr_188010003%40port_bgr&OG_MainIDs=20193&PageID=0&Avalability=true&CategoryID=Geologie&SubcategoryID=PUB-0010_0) (German Federal Agency for Geosciences and Raw Materials BGR, Hannover, 2003). <http://www.bgr.bund.de/EN/Themen/SammlungenGundlagen/GG_geol_Info/Europa/IGME5000/igme5000_inhalt_en.html?nn=1556480>
14. German Federal Agency for Geosciences and Raw Materials BGR. Geological Map of Germany, Scale 1:1000000, 4th edition. (BGR, Hannover 1993). <http://www.bgr.bund.de/EN/Themen/Sammlungen-Grundlagen/GG_geol_Info/Karten/Deutschland/GK1000/gk1000_inhalt_en.html>
15. Kirkham, D. & Bartholomew, W. V. Equations for following nutrient transformations in soil utilizing tracer data. *Soil Sci. Soc. Am. Proc.* **18**, 33-34 (1954).
16. Wu, H., et al. Feedback of grazing on gross rates of N mineralization and inorganic N partitioning in steppe soils of Inner Mongolia. *Plant Soil*, **340**, 127–139 (2011)
17. Stark, J.M. [Nutrient Transformations.](http://www.biology.usu.edu/files/uploads/Faculty/Stark-J/Stark2000-Methods%20in%20Ecosystem%20Science.pdf) Pages 215-234 in O.E. Sala, R.B. Jackson, H.A. Mooney, and R. Howarth (eds.), Methods in Ecosystem Science (Springer, New York, 2000).
