# Supplementary material for: Climate Change Impairs Nitrogen Cycling in European Beech Forests
Source: PLoS One. 2016 Jul 13;11(7):e0158823. doi: 10.1371/journal.pone.0158823 (PMC4943676; doi:10.1371/journal.pone.0158823)
Supplement: S5 Table — (DOCX) [file pone.0158823.s007.docx]

**S5 Table. Aboveground and belowground dry plant biomass (mg) of beech seedlings for the three harvest dates.**

|  |  | **June** | **August** | **September** |
| --- | --- | --- | --- | --- |
| **Aboveground** | NW | 2325±134 | 2590±155 | 1919±93 |
|  | SW | 2031±106 | 2065±90 | 1762±98 |
| **Belowground** | NW | 1893±98 | 2421±155 | 2789±126 |
|  | SW | 1546±7 | 1576±60 | 1955±96 |

n=48 (June and August); n=24 (September). Significantly larger biomass at NW compared to SW tested for single harvest dates is indicated by dark blue colour.
