# Supplementary material for: Climate Change Impairs Nitrogen Cycling in European Beech Forests
Source: PLoS One. 2016 Jul 13;11(7):e0158823. doi: 10.1371/journal.pone.0158823 (PMC4943676; doi:10.1371/journal.pone.0158823)
Supplement: S6 Table — (DOCX) [file pone.0158823.s008.docx]

**S6 Table. ^13^C recovery (mg ^13^C excess, and % of ^13^C excess applied via glutamine) in plant (sum of fine roots, coarse roots, stem, leaves) for the single harvesting dates (month_time after glutamine labelling).**

|  | **Mean**  **[mg ^13^C excess]** | **SD** | **% of applied**  **^13^C excess** | **Mean**  **[mg ^13^C excess]** | **SD** |
| --- | --- | --- | --- | --- | --- |
| **June_6h** | 0.0072 | 0.0078 | 0.4280 | -0.0028 | 0.0031 |
| **June_48h** | -0.0006 | 0.0032 | -0.0347 | -0.0127 | 0.0082 |
| **August_6h** | 0.0003 | 0.0056 | 0.0194 | -0.0007 | 0.0031 |
| **August_48h** | 0.0002 | 0.0048 | 0.0108 | 0.0005 | 0.0043 |
| **September_3months** | 0.0248 | 0.0086 | 1.4719 | 0.0117 | 0.0112 |

No significant differences were observed between NW and SW.
