# Supplementary material for: Climate Change Impairs Nitrogen Cycling in European Beech Forests
Source: PLoS One. 2016 Jul 13;11(7):e0158823. doi: 10.1371/journal.pone.0158823 (PMC4943676; doi:10.1371/journal.pone.0158823)
Supplement: S7 Table — (DOCX) [file pone.0158823.s009.docx]

**S7 Table. ^13^C enrichment (atom%) in excess of natural abundance in mycorrhizal root tips harvested in June, August and September.**

| **Harvest date_time after ^13^C labelling** | NW | | SW | | Two-Way-ANOVA | | |
| --- | --- | --- | --- | --- | --- | --- | --- |
|  | mean | SE | mean | SE |  | p | F |
| **June_6h** | -0.0004 | 0.0001 | -0.0006 | 0.0003 | Exposure | 0.924 | 0.009 |
| **June_48h** | -0.0002 | 0.0002 | -0.0014 | 0.0003 | Time | **0.000** | 6.178 |
| **August_6h** | 0.0005 | 0.0006 | 0.0000 | 0.0005 | Interactions | **0.003** | 4.602 |
| **August_48h** | -0.0010 | 0.0003 | -0.0007 | 0.0004 |  |  |  |
| **September_3 months** | -0.0003 | 0.0005 | 0.0012 | 0.0002 |  |  |  |

No significant ^13^C excess enrichment was observed in mycorrhiza within 48 hours after ^13^C glutamine labelling.
